# Dosage effect of multiple genes accounts for multisystem disorder of myotonic dystrophy type 1

**DOI:** 10.1038/s41422-019-0264-2

**Published:** 2019-12-18

**Authors:** Qi Yin, Hongye Wang, Na Li, Yifu Ding, Zhenfei Xie, Lifang Jin, Yan Li, Qiong Wang, Xinyi Liu, Liuqing Xu, Qing Li, Yongjian Ma, Yanbo Cheng, Kai Wang, Cuiqing Zhong, Qian Yu, Wei Tang, Wanjin Chen, Wenjun Yang, Fan Zhang, Chen Ding, Lan Bao, Bin Zhou, Ping Hu, Jinsong Li

**Affiliations:** 1 State Key Laboratory of Cell Biology, Shanghai Key Laboratory of Molecular Andrology, CAS Center for Excellence in Molecular Cell Science, Shanghai Institute of Biochemistry and Cell Biology, Chinese Academy of Sciences, University of Chinese Academy of Sciences, Shanghai, 200031 China; 2 State Key Laboratory of Cell Biology, CAS Center for Excellence in Molecular Cell Science, Shanghai Institute of Biochemistry and Cell Biology, Chinese Academy of Sciences, University of Chinese Academy of Sciences, Shanghai, 200031 China; 3 Shanghai University of Medicine and Health Sciences affiliated Jiading District Central Hospital, Shanghai Key Laboratory of Molecular Imaging, School of Medical Technology, Shanghai University of Medicine and Health Sciences, Shanghai, 201318 China; 4 College of Life Science of Shaoxing University, Shaoxing, Zhejiang 312000 China; 5 Department of Neurology and Institute of Neurology, First Affiliated Hospital, Fujian Medical University, Fuzhou, Fujian 350005 China; 6 Animal Core Facility, CAS Center for Excellence in Molecular Cell Science, Shanghai Institute of Biochemistry and Cell Biology, Chinese Academy of Sciences, University of Chinese Academy of Sciences, Shanghai, 200031 China; 7 State Key Laboratory of Genetic Engineering, Human Phenome Institute, Institutes of Biomedical Sciences, School of Life Sciences, Zhongshan Hospital, Fudan University, Shanghai, 200032 China; 8 Institute for Stem Cell and Regeneration, Chinese Academy of Sciences, Beijing, 100101 China; 9 School of Life Science and Technology, Shanghai Tech University, Shanghai, 201210 China

**Keywords:** Developmental biology, Biological techniques

## Abstract

Multisystem manifestations in myotonic dystrophy type 1 (DM1) may be due to dosage reduction in multiple genes induced by aberrant expansion of CTG repeats in *DMPK*, including *DMPK*, its neighboring genes (*SIX5* or *DMWD*) and downstream *MBNL1*. However, direct evidence is lacking. Here, we develop a new strategy to generate mice carrying multigene heterozygous mutations to mimic dosage reduction in one step by injection of haploid embryonic stem cells with mutant *Dmpk*, *Six5* and *Mbnl1* into oocytes. The triple heterozygous mutant mice exhibit adult-onset DM1 phenotypes. With the additional mutation in *Dmwd*, the quadruple heterozygous mutant mice recapitulate many major manifestations in congenital DM1. Moreover, muscle stem cells in both models display reduced stemness, providing a unique model for screening small molecules for treatment of DM1. Our results suggest that the complex symptoms of DM1 result from the reduced dosage of multiple genes.

## Introduction

Myotonic dystrophy type 1 (DM1) is a complex disease with variable pathological phenotypes, disease severities, and onset ages.^1–3^ The major symptoms include myotonia, muscle wasting, muscle weakness, cardiac conduction defects, cataracts and insulin resistance.^3^ DM1 is a genetic disease caused by the expansion of a CTG repeat in the 3′-untranslated region of the dystrophia myotonica protein kinase (*DMPK*) gene.^4–6^ Usually, with an increasing number of the repeats, respiratory failure and mental retardation can be observed in the most severe form of the disease (congenital DM1, CDM).^7–10^ A large number of studies have shown that the triple repeat expansion not only reduces the protein or mRNA levels of *DMPK* (haploinsufficiency model),^11–13^ but also alters the adjacent chromatin structure and reduces the expression of the neighboring genes,^14–17^ including downstream *SIX5*^18–22^ and upstream *DMWD*^23^ in DM1 cells or patients (local chromatin structure change model), albeit with controversial observations.^24–26^ Moreover, numerous studies have also shown that nuclear-accumulated RNA containing the expanded CUG repeats aberrantly recruits splicing regulators, such as *MBNL1*, and forms the ribonuclear aggregates (foci) in nucleus (RNA toxicity model), leading to misregulation of alternative splicing.^27–32^ Mouse models carrying one mutation in *Dmpk*, *Six5* or *Mbnl1*^33–37^ could only partially recapitulate the multisystem manifestations of DM1 patients.^3^ Moreover, the defects could only be observed in homozygous mutant mice (the affected proteins were completely absent), while the affected proteins were indeed present with a decreased level in human patients. Another set of DM1 mouse models are generated by overexpression of the CTG repeats;^38^ however, they could not fully recapitulate human phenotypes either.^38^ As an example, though the transgenic mouse model carrying human skeletal actin (*HSA*) gene and expressing ~250 untranslated CUG repeats in the muscle (*HSA*^LR^) can recapitulate multiple muscle phenotypes associated with DM1, it does not exhibit other common DM1-related symptoms, such as muscle wasting and cataract.^39^ Meanwhile, this model cannot mimic CDM symptoms.^39^ The potential reason might be that the random insertion of transgenes in chromatin does not affect the expression of *Dmwd*-*Dmpk*-*Six5* locus, which may be also involved in the development of complex symptoms of DM1.^39^ Moreover, muscle-specific overexpression of the repeats cannot mimic the state in human patients who carry the expanded repeats in whole body. Interestingly, a mouse model with an expanded human (CTG)_84_ repeat inserted in the endogenous *Dmpk* gene displays no overt outward anomalies,^40^ suggesting that species-specific chromatin structure at *Dmwd*-*Dmpk*-*Six5* locus may result in different expression states of local genes, leading to distinct phenotypes in human and mouse. Taken together, mouse DM1 models that better recapitulate varieties of human symptoms are required to fully understand the underlying mechanism of DM1.

The multisystem symptoms of DM1 may be caused by the combination of different mechanisms, with down-regulation of multiple genes, including *DMWD*-*DMPK*-*SIX5* and *MBNL1*.^41,42^ However, the direct evidence to support this notion is missing due to challenges to generate mice carrying multiple gene mutations simultaneously. It is time- and labor-consuming to generate mice carrying triple or quadruple mutations using conventional methods. Recently, mouse androgenetic haploid embryonic stem cells (AG-haESCs) have been successfully developed as sperm replacement to efficiently produce semi-cloned (SC) animals by injection into oocytes (intracytoplasmic AG-haESC injection, ICAHCI).^43–45^ AG-haESCs enable efficient and stable one-step generation of mice with multiple heterozygous mutant genes by ICAHCI of haploid cells carrying these mutations,^43^ allowing production of sufficient numbers of SC offspring for analyses in one generation. Thus, the haploid ESC-mediated semi-cloning technology may provide an ideal tool to generate mouse models with multiple heterozygous mutant genes in one step to mimic the reduced expression of multiple genes in human complex diseases.

In this study, we tested our hypothesis by generating triple and quadruple heterozygous mutant mice in one step through injection of haploid ESCs carrying triple or quadruple mutant genes (*Dmpk*, *Six5* and *Mbnl1 or Dmpk*, *Six5*, *Mbnl1* and *Dmwd*) into oocytes and comparing these mutant mice with wild-type (WT) SC mice. Mice with triple mutations exhibited most of the major pathogenic phenotypes observed in adult-onset DM1 patients. Mice with quadruple mutations could mimic symptoms of patients from the most severe form of DM1, CDM. Interestingly, differentiation of muscle stem cells (MuSCs) is defective in both models due to stemness reduction, which recapitulates the defects in human DM1 patients. These MuSCs provide a novel system for screening drugs to treat muscle problems in DM1 in the future.

## Results

### Generation of a novel DM1 model carrying mutations in *Dmpk*, *Six5* and *Mbnl1*

We first examined the feasibility of semi-cloning technology to generate mouse models of DM1 carrying a single mutant gene by injection of haploid cells carrying a mutation in *Dmpk*, *Six5* or *Mbnl1*, the three well-studied DM1-related genes. A haploid cell line (termed *H19*^*ΔDMR*^*-IG*^*ΔDMR*^-AGH or O48) that has been reported to efficiently support SC mouse generation^43^ was used in this study. We generated haploid ESC lines carrying mutant *Dmpk*, *Six5* or *Mbnl1* (referred to as ΔDmpk-O48, ΔSix5-O48 and ΔMbnl1-O48, respectively) (Supplementary information, Figs. S1–3) and found that these cells efficiently supported the generation of live SC pups carrying heterozygous mutant *Dmpk*, *Six5* or *Mbnl1* by ICAHCI (Supplementary information, Table S1). Mutant SC mice grew up normally to adulthood and showed similar growth curves to those of WT SC mice produced from O48 (Supplementary information, Figs. S1–3). Histological analysis of adult mice (4-6-month old) showed no obvious phenotypic abnormality in the muscle of *Dmpk*^+/−^, *Six5*^+/−^ or *Mbnl1*^+/−^ SC mice (Supplementary information, Figs. S1–3).

**Fig. 1:**
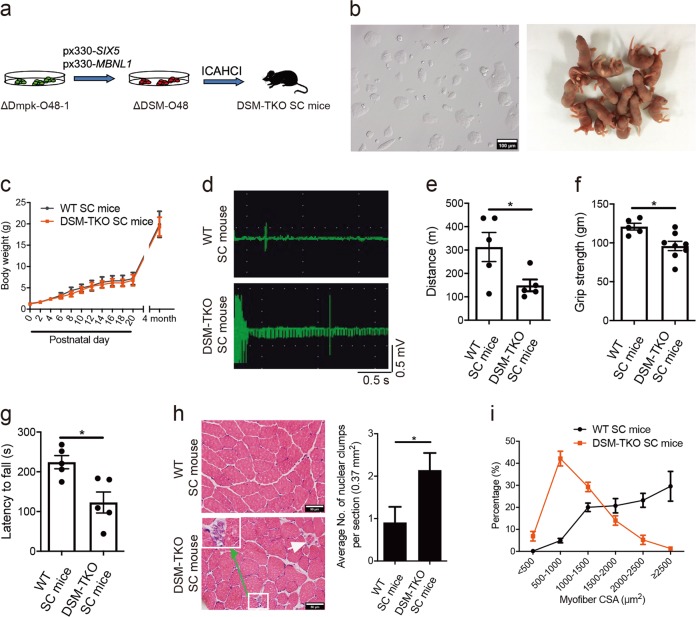
SC mice carrying heterozygous mutations in *Dmpk*, *Six5* and *Mbnl1* exhibit typical DM1-associated muscle defects. **a** Diagram of DSM-TKO SC mice generated by ICAHCI of ΔDSM-O48 cells carrying mutatnt *Dmpk*, *Six5* and *Mbnl1*. **b** Images of cultured ΔDSM-O48-1 haploid ESCs and newborn SC pups from ΔDSM-O48-1 cells. Scale bar, 100 μm. **c** Body weight analysis of DSM-TKO and WT SC mice (*n* > 5 per group, means ± SD). **d** EMG analysis of TKO SC mice (WT SC mice, *n* = 3; DSM-TKO SC mice, *n* = 2, 4-month old). **e-g** Muscle weakness of DSM-TKO SC mice was determined by treadmill test (**e**) (*n* = 5 per group, 4-month old), forelimb grip strength test (**f**) (WT SC mice, *n* = 5; DSM-TKO SC mice, *n* = 8, 4-month old) and rotarod test (**g**) (*n* = 5 per group, 4-month old). Unpaired Student’s *t*-test, *P < 0.05. **h** H&E staining of TA muscles from DSM-TKO and WT SC mice, DSM-TKO mice showing nuclear clump (green arrow) and atrophic fiber (white arrow) (WT SC mice, *n* = 11; DSM-TKO SC mice, *n* = 7, 4-month old). Unpaired Student’s *t*-test, *P < 0.05. Scale bars, 50 μm. **i** TA myofiber CSA analysis (*n* = 3 per group, 4-month old)

We then set out to generate SC mice carrying triple mutations of *Dmpk*, *Six5* and *Mbnl1* in one step using ICAHCI. We disrupted *Six5* and *Mbnl1* in ΔDmpk-O48-1 cells that have been analyzed as shown in Supplementary information, Fig. S1 and generated stable haploid cell lines carrying triple knockouts (termed ΔDSM-O48) (Fig. 1a, b). Whole-genome sequencing analysis showed no off-target effect in ΔDSM-O48-1 cells (Supplementary information, Table S2). ICAHCI experiments showed that ΔDSM-O48 cells (from two cell lines, i.e., ΔDSM-O48-1 and ΔDSM-O48-2) could reproducibly produce live SC pups (termed DSM-TKO SC mice) after injection into oocytes (Fig. 1b; Supplementary information, Fig. S4a and Table S1). Over 90% of DSM-TKO SC pups grew up to adulthood and showed similar growth profiles to those of WT SC mice (Fig. 1c and Supplementary information, Table S1). We then examined muscle phenotypes commonly observed in DM1 patients, including myotonia, muscle weakness and muscle wasting, in DSM-TKO SC mice. Electromyography (EMG) test in skeletal muscle demonstrated myotonia in adult DSM-TKO SC mice (Fig. 1d). Mouse treadmill assay and grip strength test revealed that DSM-TKO SC mice displayed muscle weakness (Fig. 1e, f); and rotarod test indicated that DSM-TKO SC mice exhibited severe motor defects (Fig. 1g). Histological analysis of tibialis anterior (TA) muscles from DSM-TKO SC mice revealed several main histological hallmarks of muscles from DM1 patients, including increased number of nuclear clump and decreased fiber size (shown as the myofiber cross-sectional area (CSA), a sign of muscle wasting) (Fig. 1h, i). Meanwhile, consistent with the clinical observations, dystrophin (Dys) expression in DSM-TKO SC mice was normal (Supplementary information, Fig. S4b), while fiber type analysis indicated an increased ratio of type I (slow) myofiber and a decreased CSA of type I in DSM-TKO SC mice (Supplementary information, Fig. S4c). Abnormalities of diaphragm muscle and small intestine were also observed (Supplementary information, Fig. S4d, e), implying the potential breathing and digestive dysfunctions in DSM-TKO SC mice. We next analyzed the cardiac structure and function in adult DSM-TKO SC mice because heart abnormalities are common in DM1 patients.^46^ Echocardiography (ECG) did not show obvious structural and functional abnormalities in 4-month-old DSM-TKO SC mice (Supplementary information, Fig. S4f, g). However, 12-month-old DSM-TKO SC mice exhibited a significantly lower ejection fraction (Supplementary information, Fig. S4g), probably induced by increased myocardial fiber abnormalities in DSM-TKO SC mice (Supplementary information, Fig. S4h).

These data demonstrate that *Dmpk*^+/−^; *Six5*^+/−^; *Mbnl1*^+/−^ SC mice can be generated in one step using haploid cells carrying triple mutations. DSM-TKO SC mice mimic the phenotype of reduced dosage of three genes and display more severe pathological consequences compared to DM1 models carrying single homozygous mutation (Supplementary information, Table S3), providing a new model of DM1. However, DSM-TKO SC mice cannot mimic several phenotypes frequently observed in DM1 patients, such as cataracts and CDM symptoms, implying that other genes may be involved.

### *DMWD* is involved in DM1

**Fig. 2:**
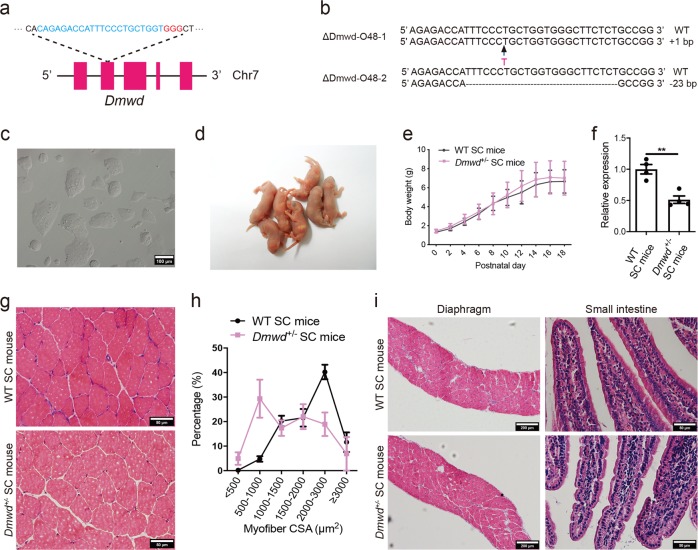
Generation of *Dmwd*^*+/*−^ SC mice through ICAHCI of haploid cells carrying mutant *Dmwd*. **a** Schematic of the sgRNA targeting *Dmwd*. **b** Sequences of the *Dmwd* gene in two cell lines (ΔDmwd-O48-1 and ΔDmwd-O48-2) carrying CRISPR-Cas9-induced gene modifications. **c** Phase-contrast image of ΔDmwd-O48-1 cell line. Scale bar, 100 μm. **d** Newborn SC pups generated from ΔDmwd-O48-1 cells. **e** Average body weight of *Dmwd*^*+/*−^ SC mice and WT SC mice (*n* > 8 per group, means ± SD). **f** Transcription analysis of *Dmwd* in TA muscles showed that the transcription level of *Dmwd* was significantly lower in *Dmwd*^*+/*−^ SC mice compared with WT SC mice (*n* = 4 per group, 4-6-month old). Unpaired Student’s *t*-test, **P < 0.01. **g** Representative images of H&E staining of TA muscles from *DMWD*^*+/*−^ SC mice and WT SC mice (4-6-month old). Scale bars, 50 μm. **h** CSA analysis of TA muscle showed muscle wasting in *DMWD*^*+/*−^ SC mice (*n* = 3 per group, 4-6-month old). **i** Representative images of H&E staining showed normal histological structure of diaphragm and small intestine in *DMWD*^*+/*−^ SC mice (4-6-month old). Scale bars, 200 µm for diaphragm; 50 µm for small intestine

It has been shown that expansion of the CTG repeats in *DMPK* produces allele-specific effects on transcription of the two adjacent genes, *SIX5* (downstream of *DMPK*) and *DMWD* (upstream of *DMPK*) through changes of the local chromatin structure, leading to down-regulation of *SIX5* and *DMWD* in DM1 patients.^14,18–21,23,47–51^ The effects of *SIX5* in DM1 have been well characterized using *Six5*-knockout mice;^35,36,52^ however, the role of *DMWD* in DM1 has not been analyzed yet. We set out to test the function of *Dmwd* by generating haploid cells carrying mutant *Dmwd* gene using CRISPR-Cas9 (Fig. 2a).^53^ Ten stable cell lines carrying mutant *Dmwd* gene (termed ΔDmwd-O48) were obtained (Fig. 2b, c). By injecting these cells into oocytes, live SC pups could be efficiently produced (Fig. 2d and Supplementary information, Table S1). *Dmwd*^+/−^ SC mice grew up to adulthood normally (Fig. 2e, f; Supplementary information, Table S1). Phenotype analysis showed that the myofiber CSA was dramatically reduced in adult *Dmwd*^+/−^ mice (Fig. 2g, h), suggesting that *DMWD* is involved in pathological mechanism of DM1. However, other symptoms of DM1, such as cardiomyocyte defects, cataracts, and abnormalities of diaphragm muscle and small intestine (Fig. 2i), cannot be observed in *Dmwd*^+/−^ mice, suggesting that *DMWD* is not sufficient to account for all the complex multisystem symptoms of DM1.

### Mice carrying quadruple mutations show typical symptoms in CDM patients

**Fig. 3:**
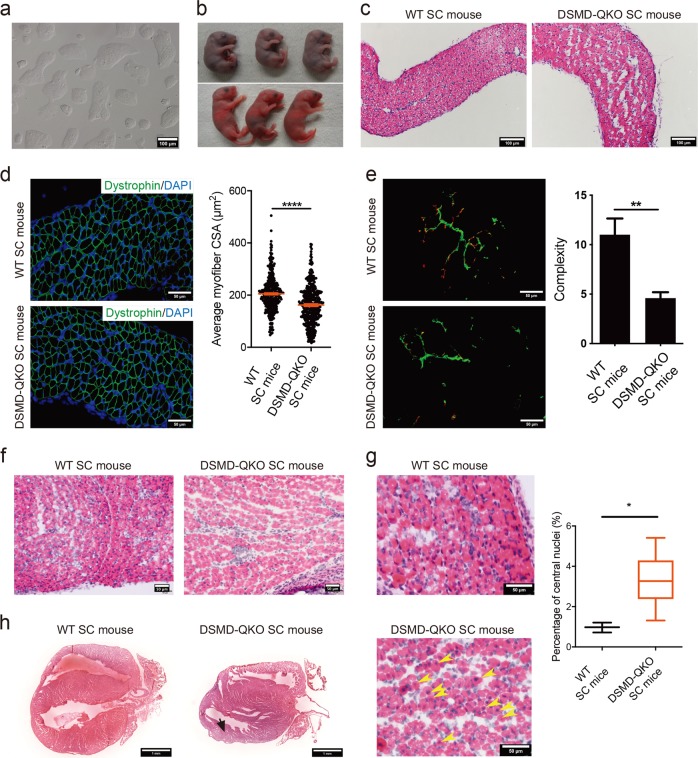
SC mice carrying heterozygous mutations in *Dmpk*, *Six5*, *Mbnl1* and *Dmwd* show CDM phenotypes. **a** Phase-contrast image of cultured ΔDSMD-O48-1 haploid cells. Scale bar, 100 μm. **b** 23% of newborn DSMD-QKO SC pups died during perinatal period (upper), while others survived (lower). **c** H&E staining of diaphragm sections from DSMD-QKO and WT SC pups (P1). Scale bars, 100 μm. **d** Diaphragm CSA analysis of DSMD-QKO and WT SC mice (P1) (*n* = 3 per group). Unpaired Student’s *t*-test, ****P *<* 0.0001. Scale bars, 50 μm. **e** Immunofluorescent staining of bungarotoxin (red) and neurofilament H (green), and complexity analysis of NMJs of DSMD-QKO and WT SC mice (P1) (WT SC pups, *n* = 2; DSMD-QKO SC pups, *n* = 5). Unpaired Student’s *t*-test, **P *<* 0.01. Scale bars, 50 μm. **f** H&E staining of TA muscle sections (P1) showing less myofiber in DSMD-QKO SC mice (2/5). Scale bars, 50 μm. **g** H&E staining of TA muscle of DSMD-QKO and WT SC (P0) mice and the percentage of myofibers containing centrally located nuclei in TA muscle (*n* = 3 per group). Yellow arrows indicate the myofibers with central nuclei. Unpaired Student’s *t*-test, *P < 0.05. Scale bars, 50 μm. **h** H&E staining of heart cryo-sections (P17) showing left ventricular posterior wall attenuation in DSMD-QKO SC mice (2/7, black arrow). Scale bars, 1 mm

Next, we tested whether compound loss of *Dmpk*, *Six5*, *Mbnl1* and *Dmwd* could give rise to a mouse model recapitulating the most of the symptoms in DM1 patients and the more severe CDM manifestations. We further mutated *Dmwd* gene in ΔDSM-O48-1 cells and generated stable haploid cell lines carrying quadruple mutations (termed ΔDSMD-O48) (Fig. 3a). Off-target analysis indicated no unexpected mutation site in the tested cells (ΔDSMD-O48-2) (Supplementary information, Table S2). ICAHCI of three lines (ΔDSMD-O48-1, -2 and -3) generated *Dmpk*^+/−^; *Six5*^+/−^; *Mbnl1*^+/−^; *Dmwd*^+/−^ SC mice (DSMD-QKO SC mice) efficiently (Supplementary information, Fig. S5a, b and Table S1). In contrast to mice carrying single or triple mutations, ~22% of newborn DSMD-QKO SC pups with normal body weights died in a couple of hours after birth (Fig. 3b). The autopsy indicated that the lung expansion failure might contribute to the neonatal death of DSMD-QKO SC pups (Supplementary information, Fig. S5c). Lung expansion failure can be caused by dysfunction of diaphragm muscle. We therefore dissected diaphragm from DSMD-QKO SC pups, which showed obvious pathogenic alterations, including reduced myofiber CSA and disorganized myofibers in diaphragm (Fig. 3c, d). Moreover, immunofluorescent staining analysis showed a significant decrease of the complexity of mature neuromuscular junctions (NMJs) in DSMD-QKO diaphragm muscles (Fig. 3e). Taken together, these results indicate that our DSMD-QKO SC mouse model recapitulates the postnatal respiratory insufficiency in CDM patients, which is the main cause of neonatal mortality of CDM patients.^54^ They also suggest that the postnatal respiratory insufficiency in CDM patients may be due to the defects of diaphragm muscle.^55^

We next characterized the growth profiles of DSMD-QKO SC mice. Interestingly, DSMD-QKO SC pups exhibited growth-retarded phenotype (Supplementary information, Fig. S5d-f) and 46% of them died within 3 weeks postnatally (Supplementary information, Table S1). We sacrificed DSMD-QKO SC pups on postnatal day 2 (P2) and observed residual milk in their stomachs, excluding the possibility that they died of feeding difficulties. P2 pups exhibited overt abnormal intestines (Supplementary information, Fig. S5g), consistent with the gastrointestinal abnormalities observed in CDM patients.^54^ Meanwhile, some of the DSMD-QKO SC pups had abnormal talus bone development (Supplementary information, Fig. S5h), recapitulating the distinguishing feature of talipes in CDM patients.^56^ Neonatal hypotonia, another typical manifestation of CDM patients,^54^ was also observed in DSMD-QKO SC pups (Supplementary information, Fig. S5i), probably due to disorganized muscle fibers and less muscle mass (Fig. 3f). The percentage of myofibers containing centrally located nuclei increased in the mutant pups (Fig. 3g), which is a common symptom of DM1 reflecting constantly regenerating muscles with immature fibers.^39,41,57^ Cardiac problem is considered to be a common cause of sudden death in patients with DM1.^46,58^ We then performed ECG test and found that 2 of 7 mice (P17) exhibited ventricular premature beats (Supplementary information, Fig. S5j). Interestingly, these two pups died in a few days after ECG examination. Histological analysis of the heart showed the ventricular and atrial wall attenuation (Fig. 3h). Taken together, DSMD-QKO SC mice represent a novel model that can mimic developmental defects of CDM patients, suggesting that the reduced dosage of multiple genes is responsible for the CDM phenotypes and *DMWD* is involved in the development of DM1 symptoms.

### Adult DSMD-QKO SC mice exhibit typical DM1 phenotypes

**Fig. 4:**
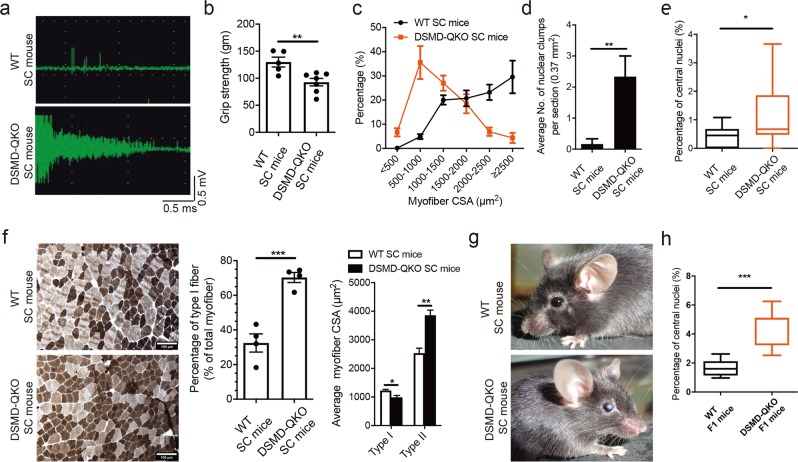
DM1-related pathogenic phenotypes in adult SC mice carrying quadruple mutations. **a** EMG analysis indicates typical waxing-waning myotonia in QKO SC mice (WT SC mice, *n* = 3; DSMD-QKO SC mice, *n* = 5, 4-6-month old). **b** Forelimb grip strength test (WT SC mice, *n* = 5; DSMD-QKO SC mice, *n* = 7, 4-6-month old). Unpaired Student’s *t*-test, **P *<* 0.01. **c** TA CSA analysis (*n* = 3 per group, 4-6-month old). **d** Nuclear clump analysis of TA muscle (WT SC mice, *n* = 6; DSMD-QKO SC mice, *n* = 3, 4-6-month old). Unpaired Student’s *t*-test, **P *<* 0.01. **e** The percentage of myofibers containing centrally located nuclei in TA muscle of QKO and WT SC mice (P30) (*n* = 3 per group). Unpaired Student’s *t*-test, *P < 0.05. **f** Representative images of ATPase staining and fiber type analysis (*n* = 4 per group, 4-6-month old). Unpaired Student’s *t*-test, *P *<* 0.05, **P *<* 0.01, ***P *<* 0.001. Scale bars, 100 μm. **g** Dust-like opacities in eyes of DSMD-QKO SC mice (3/7, 4-6-month old). **h** The percentage of myofibers containing centrally located nuclei in TA muscles of QKO and WT F1 mice (*n* = 5 per group, P0). Unpaired Student’s *t*-test, ***P < 0.001

DSMD-QKO SC mice, once survived at weaning, could grow up to adult (Supplementary information, Fig. S5d). Western blotting analysis confirmed the reduction of protein levels of DMPK, SIX5, MBNL1 and DMWD in the muscle of DSMD-QKO SC mice (Supplementary information, Fig. S6a, b). Mass spectrometry further confirmed protein reduction in adult QKO SC mice (Supplementary information, Fig. S6c). Interestingly, adult DSMD-QKO SC mice exhibited adult DM1-associated muscle symptoms, including severe myotonia, muscle weakness and motor deficits (4-6-month old) (Fig. 4a, b; Supplementary information, Fig. S7a, b). Histological analysis indicated obvious structural abnormalities in limb muscles, including muscle wasting, and increased number of nuclear clump (Fig. 4c, d; Supplementary information, Fig. S7c, d). Similar histological abnormalities could also be observed in diaphragm muscle (Supplementary information, Fig. S7f). While QKO SC mice on P30 showed a high fraction of myofibers containing central nuclei (Fig. 4e and Supplementary information, Fig. S7e), this histological abnormality was milder with ages, consistent with a phenotype in another DM1 mouse model generated by overexpression of CUGBP1.^59^ Meanwhile, fiber type analysis showed an increased percentage and a decreased CSA of type I myofibers in DSMD-QKO SC mice (Fig. 4f). Moreover, the complementary increase of type II (fast) myofiber CSA was also observed in QKO SC mice (Fig. 4f), representing another severe symptom in DM1 patients.^60^ In addition to muscle abnormalities, adult DSMD-QKO SC mice displayed other prominent DM1-associated features, such as distinct dust-like cataracts (Fig. 4g) that is similar to the symptoms of DM1 patients, overt small intestine abnormalities^54^ and endocrine dysfunction displayed by big variation of endocrine hormone levels in different individuals (Supplementary information, Fig. S7g-i), mimicking the wide variations observed in patients.^61^ Although adult DSMD-QKO SC mice did not show major heart arrhythmias, they exhibited the overt functional defects and histological abnormalities in heart (Supplementary information, Fig. S7j-k).

Since mis-splicing is a characteristic feature of DM1 and the dosage of splicing factor *Mbnl1* is also reduced in our models, we next analyzed splicing profiles of 11 DM1-related genes in P2, P10 and adult mice (4-month old). RT-qPCR analysis demonstrated the misregulation of *Ldb3*, *Serca1*, *m-TTN*, *Tmem63b*, *Sorbs1* and *Spag9* mRNA splicing, while the splicing efficiencies of *Clcn1*, *Ryr1*, *Ryr2* and *Tnnt3* mRNAs were normal (Supplementary information, Fig. S8; data not shown), reflecting the heterogeneity of mis-splicing in patients.^62,63^ A few mis-spliced genes observed in human patients appeared to be normal in our model, suggesting that other splicing factors such as MBNL2^64^ and CUGBP1^59^ might be involved in DM1.

Next, QKO F1 mice were obtained through regular mating with WT males (C57BL/6J background). Consistently, mRNA analysis showed the reduced expression of the four genes in both SC and F1 DSMD-QKO mice (Supplementary information, Fig. S9a). Interestingly, F1 QKO mice exhibited typical phenotypes shown in SC QKO mice, such as splicing abnormalities, TA and diaphragm muscle structure organization defects, and reduced muscle fiber size (Supplementary information, Fig. S9b-e). Furthermore, F1 QKO pups (P0) showed high percentage of the myofibers containing centrally located nuclei in TA muscles (Fig. 4h and Supplementary information, Fig. S9f). Taken together, DSMD-QKO SC mice can recapitulate most of the symptoms in DM1 and CDM (Supplementary information, Table S3), providing another new model for DM1 study. Furthermore, the defects can be passed to F1 of QKO SC mice, further suggesting that the DM1 symptoms caused by reduced dosage of multiple genes are stable.

### MuSCs are defective in DSM-TKO and DSMD-QKO SC mice

**Fig. 5:**
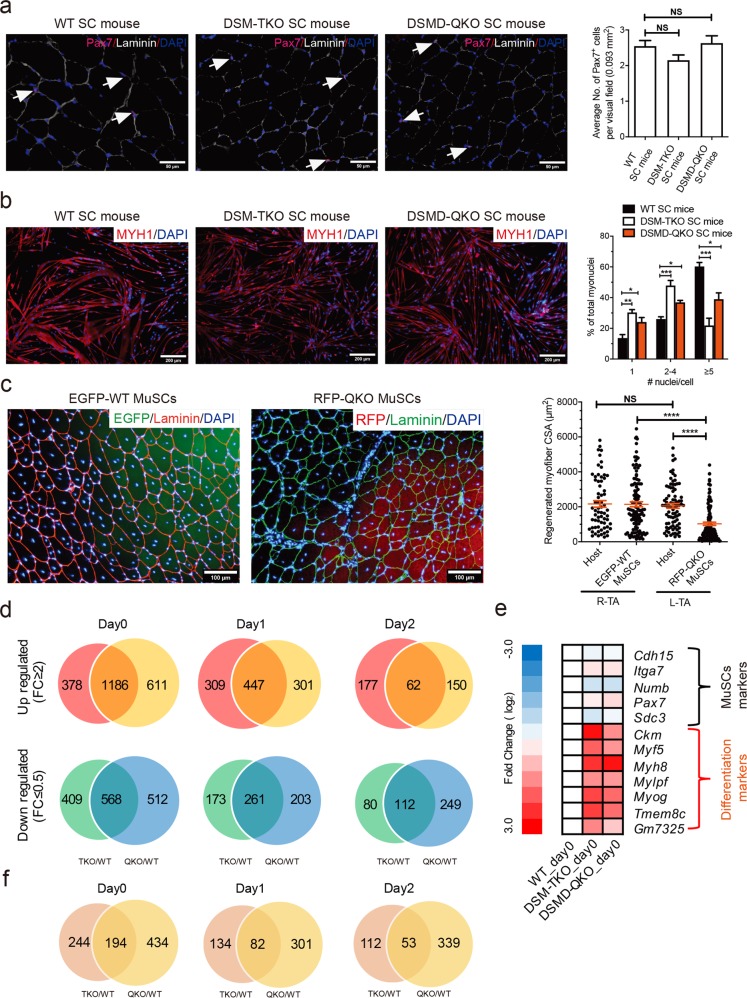
Decreased differentiation potential of MuSCs from TKO or QKO DM1 mice. **a** Representative images of PAX7 immunostaining (pink) in TA muscle sections from adult TKO and QKO mice (4-6-month old, *n* = 3 per group). White arrows indicate PAX7-positive cells. Scale bars, 50 µm. Unpaired Student’s *t*-test. NS, no significant differences. **b** In vitro differentiation of MuSCs (*n* = 3 per group, 2-month old). One-way ANOVA, *P *<* 0.05, **P *<* 0.01, ***P *<* 0.001. Scale bars, 200 μm. **c** In vivo differentiation of EGFP-WT MuSCs and RFP-QKO MuSCs (*n* = 2 per group, 2-month old). R-TA represents TA of right hind leg, while L-TA represents TA of left hind leg. One-way ANOVA, ****P *<* 0.0001. NS, no significant differences. Scale bar, 100 μm. **d** Venn diagrams show that the number of common DEGs between mutant and WT samples dramatically decreased following MuSC differentiation (from Day 0 to Day 2). **e** Heat map of stem cell markers and differentiation markers in DM1 and WT MuSCs. **f** Venn diagrams show that the number of common mis-splicing genes (skipped exon events) between mutant and WT samples dramatically decreased following MuSC differentiation (from Day 0 to Day 2)

Previous studies have shown defects in MuSCs of DM1 patients, including aberrant stemness,^65,66^ abnormal proliferation^65,67,68^ and defective differentiation;^65,69,70^ we thus characterized the MuSCs in TKO and QKO SC mice. Immunofluorescent staining of PAX7, the MuSC marker, indicated similar numbers of MuSCs in TKO, QKO (4-6-month old) and control SC mice at the same age (Fig. 5a). FACS analysis further confirmed that the ratio of MuSCs (CD34^+^:integrin-α_7_^+^:CD31^−^:CD45^−^:CD11b^−^:Sca1^−^)^71^ were not affected in both DM1 models (Supplementary information, Fig. S10a). We then tested the in vitro proliferation ability of TKO and QKO MuSCs using the long-term expansion protocol established by Fu et al.^72^. The results showed that the proliferation abilities of both types of MuSCs were comparable to that from WT SC mice (Supplementary information, Fig. S10b, c).

We next analyzed the differentiation potentials of TKO and QKO MuSCs by culturing them in differentiation medium as reported previously.^72^ The results showed that the fusion index decreased in myofibers differentiated from both TKO and QKO MuSCs (Fig. 5b). In contrast, MuSCs from a well-established transgenic mouse model expressing long repeat of CUG (*HSA*^LR^), which develops myotonia and myopathy,^39^ did not show differentiation defects in vitro (Supplementary information, Fig. S10c-e). To further confirm that the differentiation defect was cell autonomous, we performed MuSC transplantation experiments. We generated QKO SC mice carrying RFP transgene (termed RFP-QKO SC mice) and control SC mice carrying EGFP transgene (EGFP-WT SC mice) (Supplementary information, Fig. S11a, b) and then obtained MuSCs from the two strains, respectively. Consistent with the above results, RFP-QKO MuSCs showed normal proliferation ability and decreased in vitro differentiation potential (Supplementary information, Fig. S11c). We next transplanted RFP-QKO or EGFP-WT MuSCs to TA muscles of non-fluorescent WT recipients. The results showed that the CSA of RFP^+^ myofibers (myofibers generated from the transplanted RFP-QKO MuSCs) was significantly smaller than that of the EGFP^+^ myofibers generated from the transplanted EGFP-WT MuSCs (Fig. 5c). Since both types of MuSCs shared the same recipient microenvironment, we conclude that the differentiation defects of QKO MuSCs are cell autonomous. Together, these results demonstrate decreased differentiation potential of MuSCs in TKO and QKO SC mice, mimicking another important symptom in DM1 patients.

We next attempted to reveal the underlying mechanisms by comparing the genome-wide gene expression profiles of MuSCs (Day 0) and differentiating cells (Day 1 and Day 2) between mutant and normal SC mice (Supplementary information, Fig. S12a). Clustering analysis indicated that cells with the same differentiation stage rather than with the same genotype exhibited higher correlation (Supplementary information, Fig. S12b), suggesting that TKO and QKO mutations do not dramatically change the cell fate. Interestingly, the total number of differentially expressed genes (DEGs) between mutant and WT cells dramatically decreased after induction of differentiation (Fig. 5d), implying that the major differences existed at the stem cell stage. Enrichment analysis of DEGs on Day 0 showed that muscle differentiation-related genes (such as muscle development and contraction) were up-regulated in mutant MuSCs compared to WT cells (Fig. 5e; Supplementary information, Fig. S12c-e). In contrast, the expression levels of stem cell markers were similar (Fig. 5e; Supplementary information, Fig. S12d, e). Nevertheless, qRT-PCR analyses indicated that the expression levels of the muscle differentiation-related genes were comparable in differentiating cells from mutant and WT MuSCs (Supplementary information, Fig. S12f). We further analyzed the transcriptome-wide splicing events from RNA-seq data and found that, consistent with the results in Fig. 5d, the mis-splicing genes mainly existed in MuSCs (Fig. 5f and Supplementary information, Fig. S12g). These results demonstrate that MuSCs of TKO and QKO SC mice are at a more committed state.^73^ The premature expression of differentiation genes at stem cell stage suggests partial loss of stemness and may account for the decreased differentiation potential of mutant MuSCs in vitro and in vivo.

**Fig. 6:**
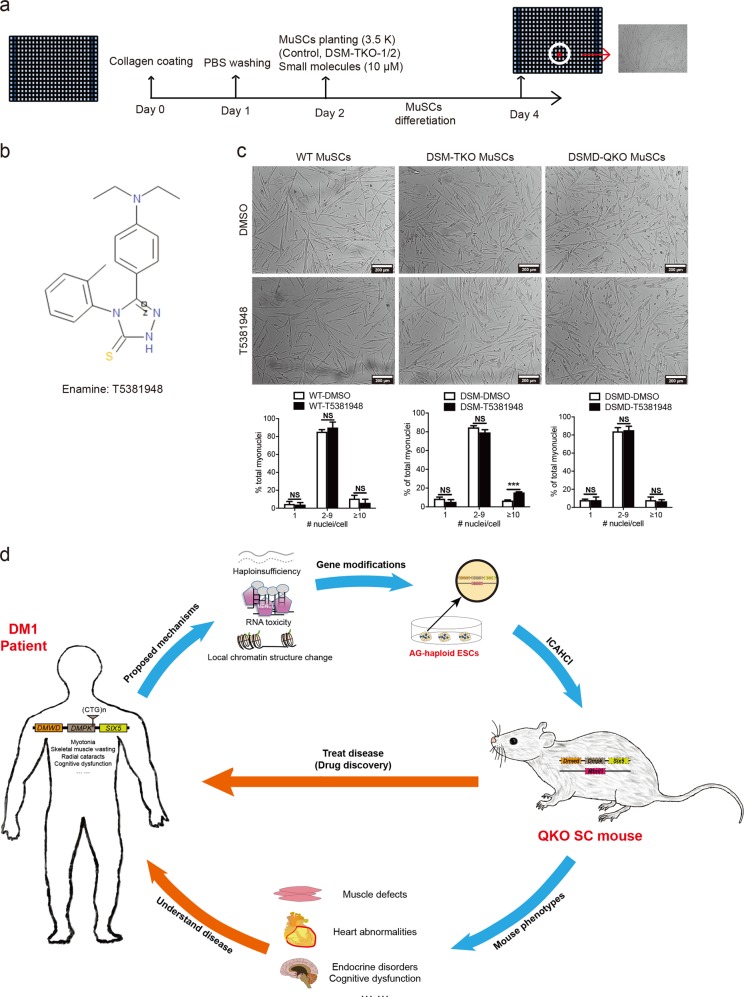
Large-scale screening and potential applications of DM1 mice. **a** Diagram of large-scale screening for small molecules that can promote differentiation of DM1 MuSCs. **b** Molecular structure of T5381948. **c** Images show that T5381948 could specifically improve the in vitro differentiation of DSM-TKO MuSCs. Scale bars, 200 μm. Unpaired Student’s *t*-test; ***P *<* 0.001; NS, no significant differences. **d** Overview of the generation and applications of DM1 mouse model with quadruple heterozygous mutations

Having shown that our DM1 models exhibit the MuSC defects that may mimic DM1 symptoms, we finally investigated whether this property could be used for screening small molecules that can be potentially used to rescue the MuSC defects in DM1 patients in future due to availability of long-term in vitro maintenance and differentiation systems of mouse MuSCs.^72^ To this end, we developed a high-throughput platform for screening small molecules that may promote MuSC differentiation, in which, the 384-well plates were coated with collagen and planted with 3,500 TKO MuSCs, followed by differentiating treatment for two days in a differentiation medium (Fig. 6a). From a total of 10,000 compounds (Medical Research Council Technology), we identified 17 positive hits that could enhance the differentiation of TKO MuSCs. Further validation assays showed that T5381948 was the most potent small molecule to specifically promote the differentiation of TKO MuSCs in vitro (Fig. 6b, c). While in vivo validation is needed in future, these results suggest that our screening system based on TKO and QKO mouse models could be useful for potential development of drugs to treat DM1.

## Discussion

Mutations or expression changes occur in multiple genes in most of the diseases, especially in those multisystem syndromes. Generation of mouse models carrying multigene mutations to faithfully mimic the dosage reduction of these genes and recapitulate the complex symptoms is the key to understand and treat these syndromes (Fig. 6d). However, it is time consuming and labor intensive to do so using the conventional methods, leading to the lack of proper animal models for many syndromes. In this proof-of-concept study, we generated mouse models with multiple heterozygous mutations, which while do not occur in patients, can mimic the reduced expression of multiple genes in human DM1 that may be the underlying mechanisms of DM1 complex symptoms, in one step by ICAHCI of haploid ESCs carrying three or four mutant genes. Interestingly, mice carrying heterozygous mutant *Dmpk*, *Six5* and *Mbnl1* (DSM-TKO SC mice) to mimic dosage reduction exhibit typical symptoms of adult-onset DM1. With the additional haploid mutation in *Dmwd*, DSMD-QKO SC mice faithfully recapitulate the pathogenic phenotypes of the more severe CDM patients. These results suggest that dosage effects of multiple genes induced by different mechanisms, including haploidinsufficiency of *DMPK*, RNA toxicity and local chromatin changes, are the major pathogenic factors in DM1 and the severity of the disease depends on the number of genes affected. In patients, longer CTG repeats are associated with more severe symptoms and earlier disease onset; however, the underlying mechanism is unknown. Our results may provide potential explanation: besides abnormal RNA-induced sequestration of splicing factor MBNL1 as suggested by the RNA toxicity model, local chromatin structure changes induced by the aberrant expansion of CTG repeats in 3′-untranslated region of *DMPK* gene first result in decreased expression of its downstream gene *SIX5* (TKO SC mice); with an increasing number of repeats, the expression of its upstream gene *DMWD* is also affected (mimicked by QKO SC mice), leading to more severe and early onset DM1 (Supplementary information, Table S3). The longer the CTG repeat is, the more genes may be affected. The wide variety of human DM1 symptoms could be due to the variant contributions of different genes, which can weigh differently in individual patients, leading to their different phenotypes. To understand more about the mechanism of DM1 will help us group the patients more accurately for better treatment in future.

Given that F1 offspring from QKO SC mice exhibit typical DM1 symptoms (Fig. 4h and Supplementary information, S9), we tried to generate stable TKO or QKO mouse lines with or without *H19*/*IG*-DMR deletions through multiple backcross with C57BL/6J mice. Surprisingly, we found that heterozygous TKO or QKO mice without *H19* and *IG*-DMR deletions (F3 and F4) did not exhibit typical DM1 symptoms. Interestingly, we unexpectedly observed upregulation of *Dmpk*, *Six5*, *Mbnl1* and *Dmwd* in the animals (data not shown), which may account for the loss of phenotypes in these strains. A recent study has shown that genetic compensation in knockout zebrafish is activated by a premature termination codon-based knockout strategy,^74^ which has been used in our study and may induce the upregulation of WT allele of the targeted genes in F3 or F4 TKO/QKO mice during normal breeding. Meanwhile, we could not exclude the possibility that the potential dosage changes of genes in *H19*/*Igf2* and *Dlk1*/*Dio3* imprinted clusters induced by *H19*-DMR and *IG*-DMR deletions in DKO-AG-haESCs may partially account for the complex symptoms of the models.^75^ Moreover, while our models can recapitulate most symptoms of DM1 patients, they do not exhibit a few typical DM1 features, such as abnormal *Clcn1* splicing, implying that other DM1-related genes including *MBNL2*^76^ and *CUGBP1*^59^ might be involved in the disease. It will be interesting to test whether mouse models with modified *Mbnl2* and/or *CUGBP1* can mimic these symptoms.

Defects in MuSCs have been shown to be related to DM1 and CDM patients.^65,67,77^ However, this pathological phenotype has not been recapitulated in mice yet until in 2017 during preparation of our manuscript, Thomas et al.^26^ reported that conditional disruption of *Mbnl3* in muscle resulted in defective MuSC differentiation. However, the expression of *MBNL3* is not affected in DM1 patients, implying that the phenotype observed in their study is not closely related to the MuSC defects observed in DM1 patients. In contrast, our TKO and QKO models generated by mimicking dosage reduction of multiple DM1-related genes in mice, exhibited defective in vitro and in vivo differentiation phenotypes, and thus might truly reflect the state in patients. Meanwhile, this property enables a unique system to screen small molecules that enhance the differentiation of DM1 MuSCs in vitro; such molecules may also improve the MuSC differentiation in vivo, ameliorating the muscle defects induced by less stemness in patients, such as muscle weakness and wasting. Taken together, our study suggests that abnormal expansion of CTG repeats in *DMPK* could disrupt the chromatin structure and affect the neighboring genes, which, combined with aberrantly expanded *DMPK* mRNA-mediated RNA toxicity mechanism, accounts for complex pathogenic phenotypes in DM1 patients.

Our method paves the road towards generation of a series of mouse models carrying multiple gene mutations in a short time to mimic different stages and severities of the complex syndromes. A potential application of the approach is to produce mice carrying multiple modifications in candidate loci that have been identified in high-throughput studies or genetic screenings to mimic clinical manifestations of multigenic diseases.^78^ In summary, we have established novel DM1 mouse models by one-step injection of haploid cells carrying three or four mutant genes into oocytes, providing suitable models to investigate the molecular mechanisms underlying complex manifestations and perform drug screening (Fig. 6d). Future analysis of the TKO and QKO mice will reveal more genes involved in the complex manifestations of DM1. Meanwhile, we hope that our haploid ESC-mediated semi-cloning technology will promote modeling of other complex diseases in mice.

## Materials and methods

### Experimental mice

All animal procedures were carried out in accordance with the guidelines of the Shanghai Institute of Biochemistry and Cell Biology (SIBCB). All mice were housed in specific pathogen-free facilities of SIBCB. Oocytes for micromanipulation were obtained from female mice of B6D2F1 (C57BL/6J × DBA/2) background. *HSA*^*LR*^ mice (FVB/n background) and *IL2r*^*−/−*^ mice (C57BL/6J background) were purchased from the Jackson Laboratory.

### Derivation of gene-modified haploid cell lines

CRISPR-Cas9-mediated gene manipulation was performed, as previously described.^53,79^ Briefly, oligos for different genes (*Dmpk*, *Six5*, *Mbnl1* and *Dmwd*) were synthesized and ligated in px330-mcherry plasmid. Constructed plasmids were then transfected into haploid cells (O48), which were cultured in the ESC medium plus 3 μM CHIR99021 and 1 μM PD0325901 (ES + 2i). The haploid cells were enriched through FACS and plated in one well of the 6-well plate at low density to obtain single-cell clone in 24 h after transfection. 6–7 days after plating, the single-cell clones were picked and separated into two parts, one for passaging and the other for sequencing to determine the genotype. For generation of EGFP-O48 and RFP-ΔDSMD-O48 haploid ESCs, the haploid ESCs were transfected with PiggBac plasmids (CAG-EGFP or CAG-RFP).

### ICAHCI

To produce SC embryos, the haploid ESCs arrested in M phase by culturing in medium containing 0.05 μg/mL demecolcine were trypsinized and resuspended in Hepes-CZB medium, then injected into oocytes using a Piezo-drill micromanipulator (Prime Technology Ltd), as described previously.^80^ The SC embryos were cultured in KSOM medium with amino acids at 37 °C under 5% CO_2_ in air. The 2-cell stage embryos were transferred into oviduct of pseudopregnant ICR females at 0.5 days post coitum with 15–20 embryos per side. Recipient mothers naturally delivered the SC pups.

### Immunostaining

Dissected tissues from diaphragm, heart and TA muscle were put into the OCT (Leica) directly, and then frozen in liquid nitrogen for 15 s. The frozen tissues could be stored in −80 °C refrigerators until sectioning. For whole heart longitudinal sections, the freshly dissected tissues were washed with PBS and fixed with 4% paraformaldehyde (PFA) at 4 °C for 30 min. After being dehydrated with 30% sucrose solution, the samples were embedded in OCT. The protocol for Pax7 (DHSB, 1:200) staining was carried out according to the previously described protocols.^72,81^ Immunostaining for dystrophin was performed using anti-dystrophin (Abcam, ab15277) as primary antibody (1:400) and Alexa 488-conjunated anti-mouse antibody (Invitrogen) as secondary antibody. All images were acquired on Leica SP8 confocal microscope. For NMJ structural analysis, the frozen transverse sections of diaphragm were stained with a-bungarotoxin conjugated with Alexa Fluor 594 (Invitrogen, B13423) for acetylcholine receptors, and then incubated with chicken ployclonal antibody for neurofilament H (EnCor Biotechnology, CPCA-NF-H, 1:2000), followed by incubation with Alexa 488-labeled secondary antibody.

### Dissection and H&E staining of mouse TA muscle

TA muscle was gently removed from the mouse hindlimb after peeling off the leg skin. After dissection, TA muscles were embedded with minimum of Tissue-Tec O.C.T compound and quickly frozen with liquid nitrogen. These tissues could be stored in −80 °C freezer before cryosectioning. Around 10 μm-thick sections were made and collected on positively charged microscope slides. Then the slides were fixed with methanol before being subjected to hematoxylin and eosin (H&E) staining. After the slides were mounted with mounting media, the images were captured with Olympus BX51 microscope. The myofiber CSA and the percentage of myofibers with central nuclei in TA muscles were analyzed with ImageJ.

### Histology

Frozen transverse sections (10 μm) of TA and diaphragm muscles were stained with H&E. Fiber CSAs were measured using ImageJ. ATPase staining was performed, as described previously.^82^ Briefly, transverse sections were incubated in pH 4.6 solution for 5–15 min, followed by staining with ATP solution. After being washed with medium containing 1% CaCl_2_ (w/v), 2% CoCl_2_ (w/v), 0.1 M Sodium Barbital solution (1:20) and 1% ammonium sulfide solution (v/v), the sections were dehydrated with ascending concentrations of alcohol. CSAs of type I and type II fibers were measured using ImageJ. For small intestine, freshly dissected tissues were flushed with PBS and 4% PFA, followed by fixation in 4% PFA overnight at 4 °C, dehydrated, embedded in paraffin, and cut at 5 μm thickness.

### Grip strength, treadmill test, rotarod test and righting assay

The forelimb grip strength of mice (4-month old) was assessed using a grip strength meter. Every mouse was tested for five times and the arithmetic average value of the records shall be as the measured value. For treadmill test, following familiarization test (6 m/min for 3 min), the mouse (4-month old) was placed on a treadmill with a start speed of 10 m/min. Running speed was increased by 2 m/min every 2 min until 30 m/min, and the distance was recorded when the mouse could no longer run. For rotarod performance test, the mouse (4-month old) was placed on a rotating rod at uniform motion (10 rpm) for 5 min and then an accelerating rotarod, which started with 4 rpm and gradually increased to 40 rpm in 5 min. The mice were pre-trained for 2 days for adaptation. The duration time on the rotarod before the mice fell off was recorded. For righting assay, the mice on P5 were placed on its back to determine whether the mice could right themselves to normal posture with four paws on the ground.

### MuSC isolation, expansion and in vitro differentiation

MuSCs were isolated according to the previously described methods.^71,72,83^ MuSCs were cultured on collagen-coated dish in T cell conditional medium for long-term expansion according to our reported protocol.^72^ Briefly, 10,000 MuSCs were seeded in a 3.5 cm dish and passaged every two days. The proliferation ability was determined during passaging period using cell counting and EdU labeling. MuSCs were differentiated in differentiation medium with 2% horse serum (Sigma). After being differentiated for 48 h, the cells were stained with anti-MYH1 antibody (Merck Millipore, 05–716), and counterstained with DAPI for counting the number of nuclei in one fiber.

### MuSC transplantation (in vivo differentiation)

MuSC transplantation was performed according to the described protocols^84,85^ with some modifications. Briefly, both hindlimbs of host *IL2r*^*−/−*^ mice were exposed to 18 Gy γ-radiations. Twenty-four hours before MuSC transplantation, 10 μM CTX (15 μL) is intramuscularly injected into mouse TA muscle via a 29 G insulin syringe. In total 5 × 10^5^ MuSCs were resuspended with 30 μL of PBS and injected into TA muscle using 29 G insulin syringe. TA muscles were collected in 4 weeks after cell transplantation and used for histological analysis.

### EdU labeling

MuSCs were cultured in MuSC medium with EdU solution (Invitrogen; final concentration, 10 μM) for 2 h at 37 °C, followed by fixation with 3.7% PFA, permealized with 0.5% Triton X-100, reacted with Click-iT reaction cocktail and counterstained with Hoechst 33342.

### Skeletal preparation and staining

Skeletal preparations were stained with Alcian blue and ARS. Briefly, the mice were executed, eviscerated and skinned. The samples were dehydrated with 95% ethanol for 3 days, and then were stained in Alcian blue solution for 3 days. After being fixed and cleared with 95% ethanol three times (1.5 h for each), the samples were treated with 2% KOH for 3–4 h and stained with ARS solution for another 3–4 h. After staining, skeletons were cleared in 1% KOH/20% glycerol and stored in 100% glycerol up to several years.

### ELISA

Insulin, PTH and TSH levels in blood were measured by ELISA kit (Ebioscience) according to the manufacturer’s instructions. The whole blood was collected from retro-orbital bleeding.

### EMG and ECG

EMG was performed under halothane anesthesia using 30-gauge concentric needle electrodes, with sampling of distal muscle group in left hindlimb. For ECG analysis, after removing the hair from chest using depilatory cream, the mouse was anesthetized and fixed on Vevo2100 (VisualSonics) by tighting the limbs on the mental detector to detect ECG. The ECG was recorded according to the manufacturer’s instructions.

### Western blotting

Dissected tissues were frozen with liquid nitrogen and stored in −80 °C refrigerators until protein extraction. Tissue homogenization was done by vortex in cell lysis buffer. After being separated on SDS-PAGE gels, the lysate was transferred to PVDF membranes and incubated with primary antibodies (anti-MBNL1, Abcam, ab108519; anti-SIX5, Abcam, ab113064; anti-DMWD, Santa Cruz, sc-167638; anti-DMPK, Santa Cruz, sc-13612) at 4 °C overnight, followed by incubation with secondary antibodies.

### RNA-seq, gene expression and alternative splicing analyses

MuSCs (Day 0) and differentiating cells (Day 1 and Day 2) were collected and subjected to RNA extraction using TRIZOL Reagent (Invitrogen). The total RNA was processed for RNA-seq by TruSeq RNA sample pre kit (Illumina) according to the manufacturer’s instructions, and examined for a RIN number (≥ 7) to inspect RNA integrity by an Agilent Bioanalyzer 2100 (Agilent Technologies). Qualified RNA was further purified by RNAClean XP Kit (Beckman Coulter, Inc.) and RNase-Free DNase Set (QIAGEN). Adaptors as well as low-quality base pairs were trimmed. RNA-seq was performed using Illumina Hiseq X TEN. Then the preprocessed reads were aligned to the protein-coding genes of mouse reference genome (NCBI Mus musculus assembly GRCm38.p5) using hisat2 (version 2.0.5) and gene expression was quantified by stringtie. Alternative splicing events were detected by Astalavista (version 3.1) and profiled using MISO based on MISO Annotations version 2.0, which were performed by Shanghai Biotechnology Corporation. Moreover, the parameter settings of MISO referred to the reference.^26^ Venn diagram was plotted in RStudio.

### RNA splicing and qRT-PCR

Total RNA was extracted from dissected tissues according to the manufacturer’s instructions. 0.5 μg of total RNA was reverse transcribed using a First Strand cDNA Synthesis kit (Toyobo). qPCR was carried out with THUNDERBIRD SYBR qPCR Mix (Toyobo) on a CFX connect Real-Time System (Bio-Rad). qPCR primers were listed in Supplementary information, Table S4. For alternative splicing analysis, the primers, which could separate the splice alternatives of target genes,^86^ were used to amplify the RNA variants, followed by electrophoresis on 2% agarose gel or SDS-PAGE to calculate the splicing efficiency (percent of spliced in).

### Small molecule screening

About 10,000 small compounds from Index Library of LifeArc (England) were screened on MuSCs planted in 384-well plates (3754) with differentiation medium. Before the compounds were added, the 384-well plate was coated with collagen and washed with PBS. 3500 MuSCs were plated by Multidrop and compounds (final concentration, 10 μM) were added by Mosquito. The MuSCs were differentiated in differentiation medium for two days and the images were captured using Operetta.

### Statistical analysis

Quantitative values are presented as means ± SEM, unless noted otherwise. Statistical differences between groups were determined by GraphPad Prism 6 using unpaired two-tailed *t*-test. No statistical method was used to predetermine sample size. Investigators were not blinded to outcome assessment.

### Accession number

All RNA-seq data sets are available through GEO under the accession number: GSE103841.

## Supplementary information

Supplementary information, Fig. S1

Supplementary information, Fig. S2

Supplementary information, Fig. S3

Supplementary information, Fig. S4

Supplementary information, Fig. S5

Supplementary information, Fig. S6

Supplementary information, Fig. S7

Supplementary information, Fig. S8

Supplementary information, Fig. S9

Supplementary information, Fig. S10

Supplementary information, Fig. S11

Supplementary information, Fig. S12

Supplementary information, Table S1

Supplementary information, Table S2

Supplementary information, Table S3

Supplementary information, Table S4
