# Supplementary material for: Dosage effect of multiple genes accounts for multisystem disorder of myotonic dystrophy type 1
Source: Cell Res. 2019 Dec 18;30(2):133–45. doi: 10.1038/s41422-019-0264-2 (PMC7015062; doi:10.1038/s41422-019-0264-2)
Supplement: Supplementary file 1 — Supplementary information, Fig. S1 [file 41422_2019_264_MOESM1_ESM.pdf]

Supplementary information, Figure S1

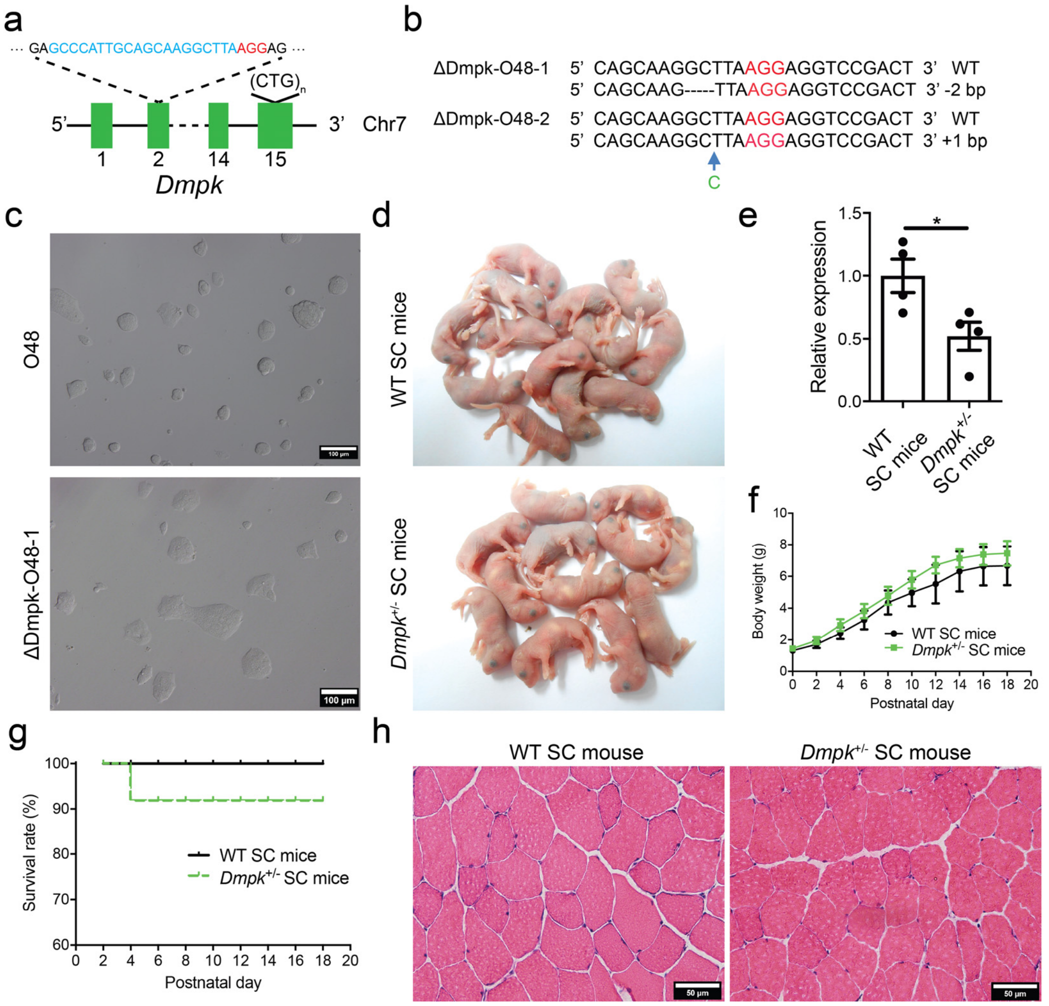

Fig. S1 Generation of *Dmpk*$^{+/-}$ SC mice through ICAHCI of haploid cells carrying mutant *Dmpk*. **a** Schematic of the sgRNA targeting *Dmpk*. **b** Sequences of the *Dmpk* gene in two cell lines (ΔDmpk-O48-1 and ΔDmpk-O48-2). **c** WT and mutant haploid cells. Scale bars, 100 μm. **d** Newborn SC pups from ΔDmpk-O48-1 cells (lower panel) and O48 cells (upper panel). **e** Transcription analysis of *Dmpk* in *Dmpk*$^{+/-}$ and WT mice ($n = 4$ per group). Unpaired Student's *t*-test, $*P < 0.05$. **f** Body weight analysis of *Dmpk*$^{+/-}$ and WT SC mice ($n > 10$ per group, means ± SD). **g** Survival curves of *Dmpk*$^{+/-}$ and WT SC mice ($n > 10$ per group). **h** Representative images of TA muscle sections from *Dmpk*$^{+/-}$ and WT SC mice. Scale bars, 50 μm.
