# Supplementary material for: Dosage effect of multiple genes accounts for multisystem disorder of myotonic dystrophy type 1
Source: Cell Res. 2019 Dec 18;30(2):133–45. doi: 10.1038/s41422-019-0264-2 (PMC7015062; doi:10.1038/s41422-019-0264-2)
Supplement: Supplementary file 2 — Supplementary information, Fig. S2 [file 41422_2019_264_MOESM2_ESM.pdf]

Supplementary information, Figure S2

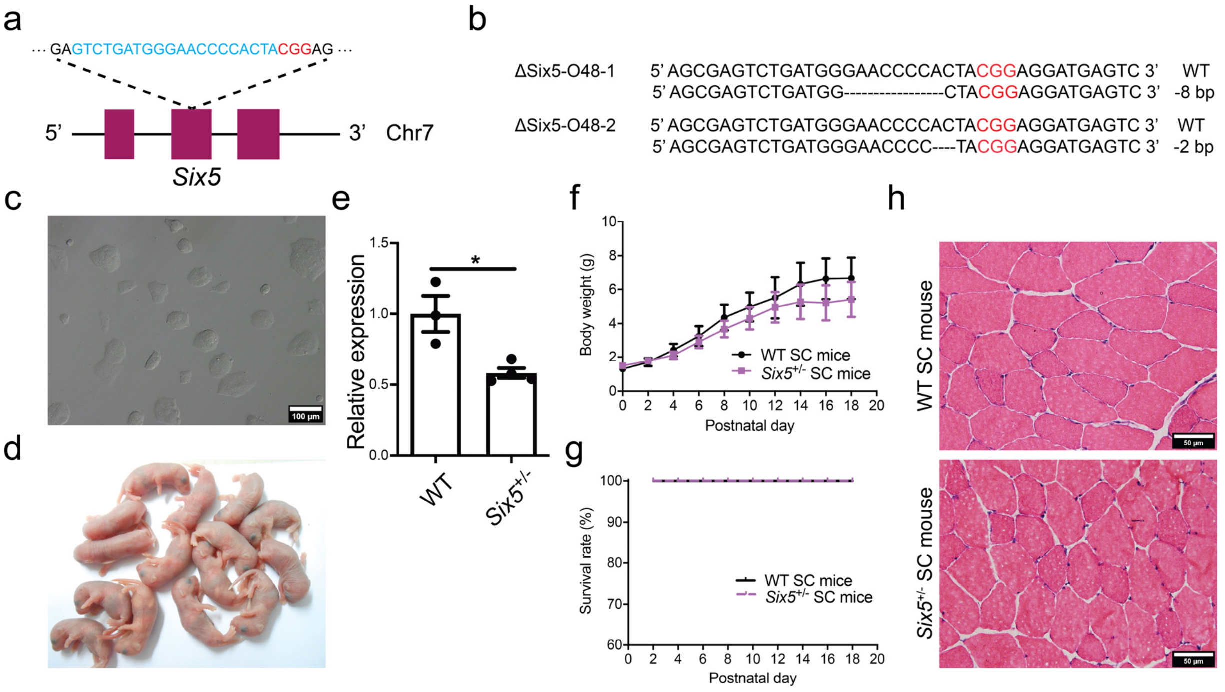

Fig. S2 Generation of *Six5*$^{+/-}$ SC mice through ICAHCI of haploid cells carrying mutant *Six5*. **a** Schematic of the sgRNA targeting *Six5*. **b** Sequences of the *Six5* gene in two mutant cell lines (ΔSix5-O48-1 and ΔSix5-O48-2). **c** Phase-contrast image of ΔSix5-O48-1 cell line. Scale bar, 100 µm. **d** Newborn SC pups generated from ΔSix5-O48-1 cells. **e** Transcription analysis of *Six5* in *Six5*$^{+/-}$ (n = 4) and WT (n = 3) SC mice. Unpaired Student's t-test, *$P < 0.05$. **f** Body weight analysis of *Six5*$^{+/-}$ and WT SC mice (n > 4 per group, means ± SD). **g** Survival curves of *Six5*$^{+/-}$ and WT SC mice (n > 4 per group). **h** Histological analysis of TA muscles from *Six5*$^{+/-}$ and WT SC mice. Scale bars, 50 µm.
