# Supplementary material for: Dosage effect of multiple genes accounts for multisystem disorder of myotonic dystrophy type 1
Source: Cell Res. 2019 Dec 18;30(2):133–45. doi: 10.1038/s41422-019-0264-2 (PMC7015062; doi:10.1038/s41422-019-0264-2)
Supplement: Supplementary file 3 — Supplementary information, Fig. S3 [file 41422_2019_264_MOESM3_ESM.pdf]

Supplementary information, Figure S3

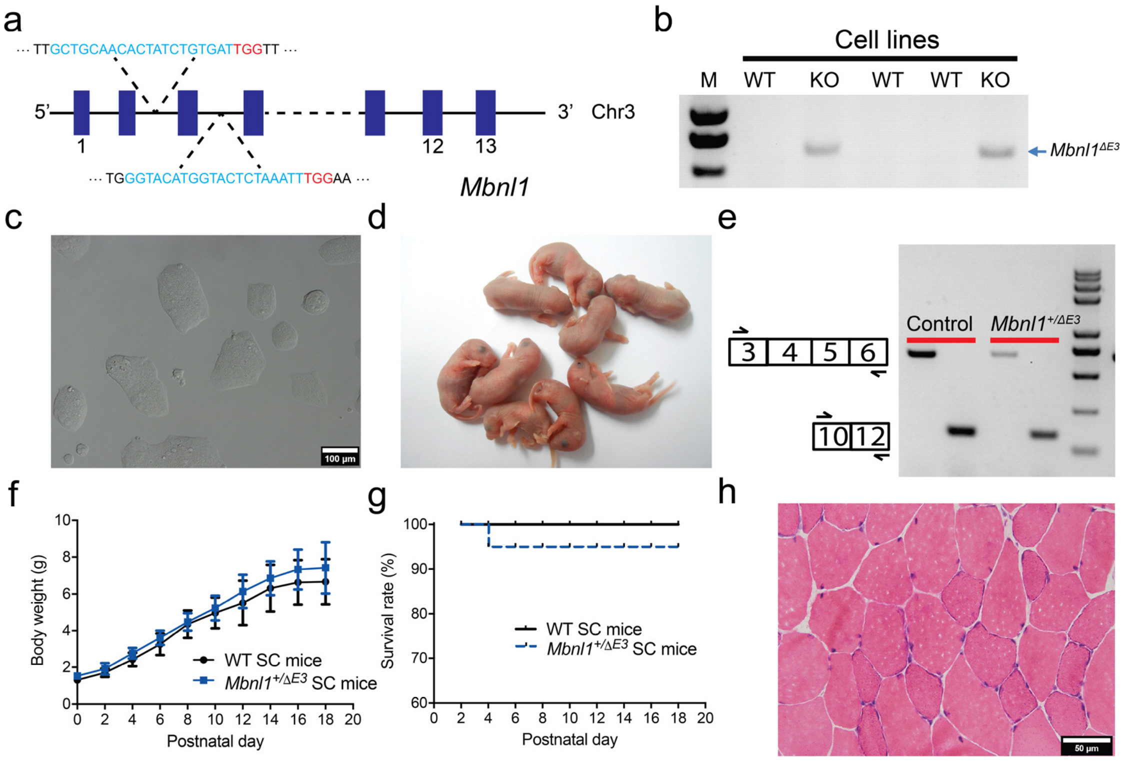

Fig. S3 Generation of *Mbnl1*$^{+/\Delta E3}$ SC mice through ICAHCI of haploid cells carrying mutant *Mbnl1*. **a** Schematic of two sgRNAs for removing exon 3 of *Mbnl1*. **b** Genotyping analysis of *Mbnl1*$^{\Delta E3}$-O48 cell lines. **c** Phase-contrast image of *Mbnl1*$^{\Delta E3}$-O48-2 cells. Scale bar, 100 µm. **d** Newborn SC pups generated from *Mbnl1*$^{\Delta E3}$-O48-2 cells. **e** RT-PCR of *Mbnl1* in skeletal muscles from *Mbnl1*$^{+/\Delta E3}$ and WT SC mice. **f** Body weight analysis of *Mbnl1*$^{+/\Delta E3}$ and WT SC mice ($n > 8$ per group, means ± SD). **g** Survival curves of *Mbnl1*$^{+/\Delta E3}$ and WT SC mice ($n > 8$ per group). **h** Representative image of H&E staining of TA muscles from *Mbnl1*$^{+/\Delta E3}$ SC mouse. Scale bar, 50 µm.
