# Supplementary material for: Dosage effect of multiple genes accounts for multisystem disorder of myotonic dystrophy type 1
Source: Cell Res. 2019 Dec 18;30(2):133–45. doi: 10.1038/s41422-019-0264-2 (PMC7015062; doi:10.1038/s41422-019-0264-2)
Supplement: Supplementary file 4 — Supplementary information, Fig. S4 [file 41422_2019_264_MOESM4_ESM.pdf]

Supplementary information, Figure S4

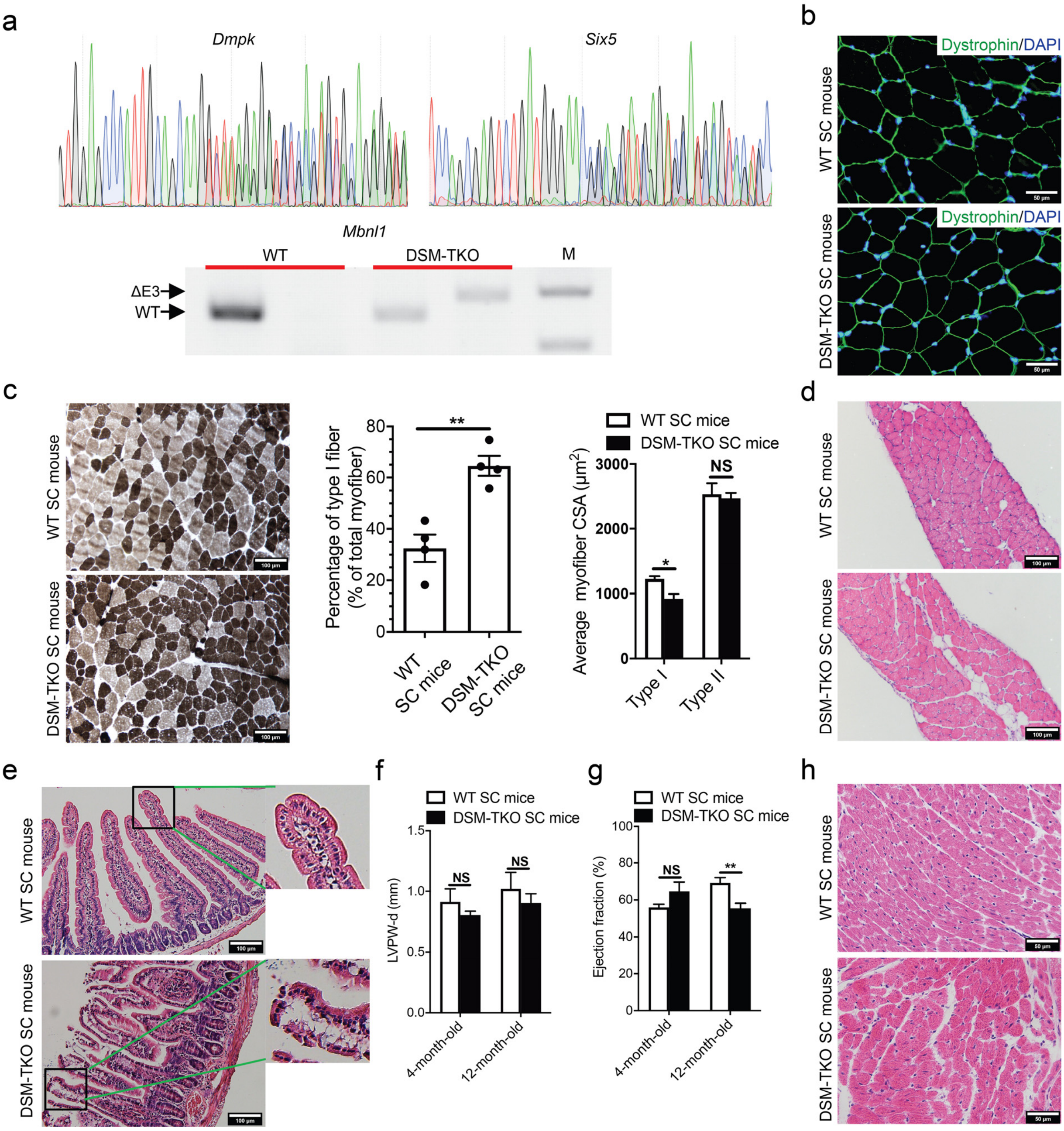

Fig. S4 DM1-associated phenotypes in adult DSM-TKO SC mice. **a** Genotyping analysis of TKO SC mice. **b** Immunofluorescent staining of dystrophin (green) in DSM-TKO and WT SC mice. Scale bars, 50 µm. **c** ATPase staining showing the increased ratio and reduced CSA of type I myofiber (darker cells) in TKO SC mice (n = 4 per group). Unpaired Student's t-test, $**P <$

0.01. NS, no significant differences. Scale bars, 100 µm. **d** H&E staining of diaphragm showing increased fat cell invasion in TKO SC mice. Scale bars, 100 µm. **e** Histological analysis of small intestine. Boxes in left panels are magnified in right panels, showing disorganization of intestine villi in TKO SC mice. Scale bars, 100 µm. **f** IVPW-d thickness (diastole) of DSM-TKO and WT SC mice at different ages ($n = 5$ per group). IVPW: left ventricular posterior wall. Unpaired Student's *t*-test. NS, no significant differences. **g** Reduced ejection fraction in 12-month-old DSM-TKO SC mice ($n = 5$ per group). Unpaired Student's *t*-test, $^{**}P < 0.01$. NS, no significant differences. **h** Myocardial disarray shown in the 12-month-old TKO SC mouse. Scale bars, 50 µm.
