# Supplementary material for: Dosage effect of multiple genes accounts for multisystem disorder of myotonic dystrophy type 1
Source: Cell Res. 2019 Dec 18;30(2):133–45. doi: 10.1038/s41422-019-0264-2 (PMC7015062; doi:10.1038/s41422-019-0264-2)
Supplement: Supplementary file 5 — Supplementary information, Fig. S5 [file 41422_2019_264_MOESM5_ESM.pdf]

Supplementary information, Figure S5

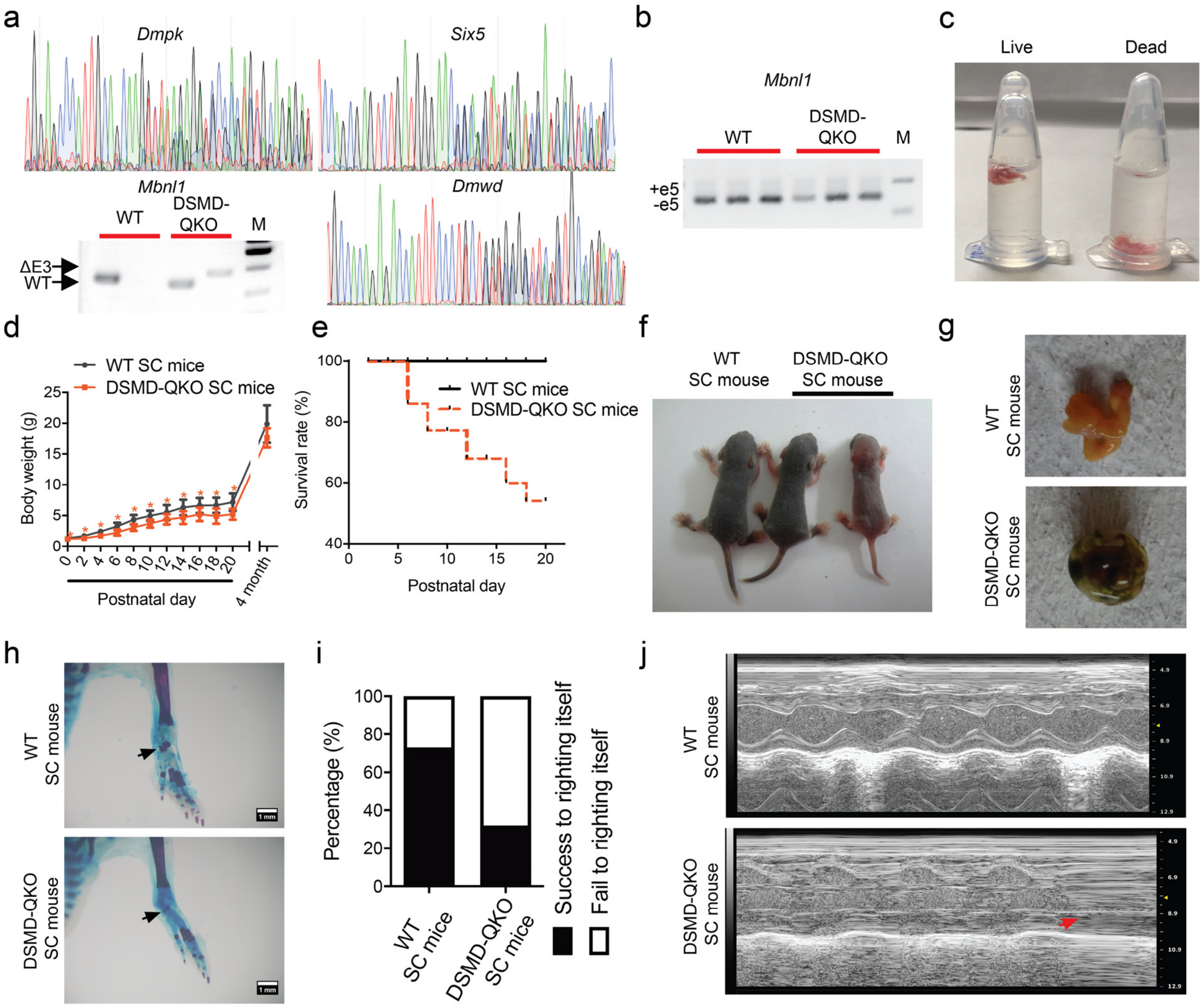

Fig. S5 CDM1-related phenotypes exhibited in DSMD-QKO SC mice before weaning. **a** Genotyping analysis of DMSD-QKO SC mice. **b** Alterative splicing of *Mbnl1* exon5 in DSMD-QKO SC mice and WT SC mice. **c** The lung dissected from dead or live DSMD-QKO SC pups in water (n = 3 per group). The lung of the dead DMSD-QKO SC pup never inflated with air sinks to the bottom (right). **d** Body weight analysis of DSMD-QKO and control SC mice revealing delayed growth in DSMD-QKO SC mice (n > 10 per group). Unpaired Student's t-test, *P < 0.05. **e** Survival curves of DSMD-QKO and WT SC mice (n > 10 per group). **f** The image

of DSMD-QKO and WT SC pups on postnatal day 5 (P5). The right one is a QKO pup with growth-retarded phenotype. The middle one is a QKO pup with normal growth. The left one is a control SC pup. **g** Developmental failure of intestines (3/16) in DSMD-QKO SC pups. **h** Smaller talus bone (3/7, black arrows) in DSMD-QKO SC pups. Scale bar, 1.0 mm. **i** Righting assay showing that more QKO SC mice (P5) failed to righting itself than control SC mice (WT SC mice, n = 15; QKO SC mice, n = 22). **j** Echocardiography experiments showing ventricular premature beats in QKO mice (2/7). Red arrow indicates the ventricular premature beat.
