# Supplementary material for: Dosage effect of multiple genes accounts for multisystem disorder of myotonic dystrophy type 1
Source: Cell Res. 2019 Dec 18;30(2):133–45. doi: 10.1038/s41422-019-0264-2 (PMC7015062; doi:10.1038/s41422-019-0264-2)
Supplement: Supplementary file 6 — Supplementary information, Fig. S6 [file 41422_2019_264_MOESM6_ESM.pdf]

Supplementary information, Figure S6

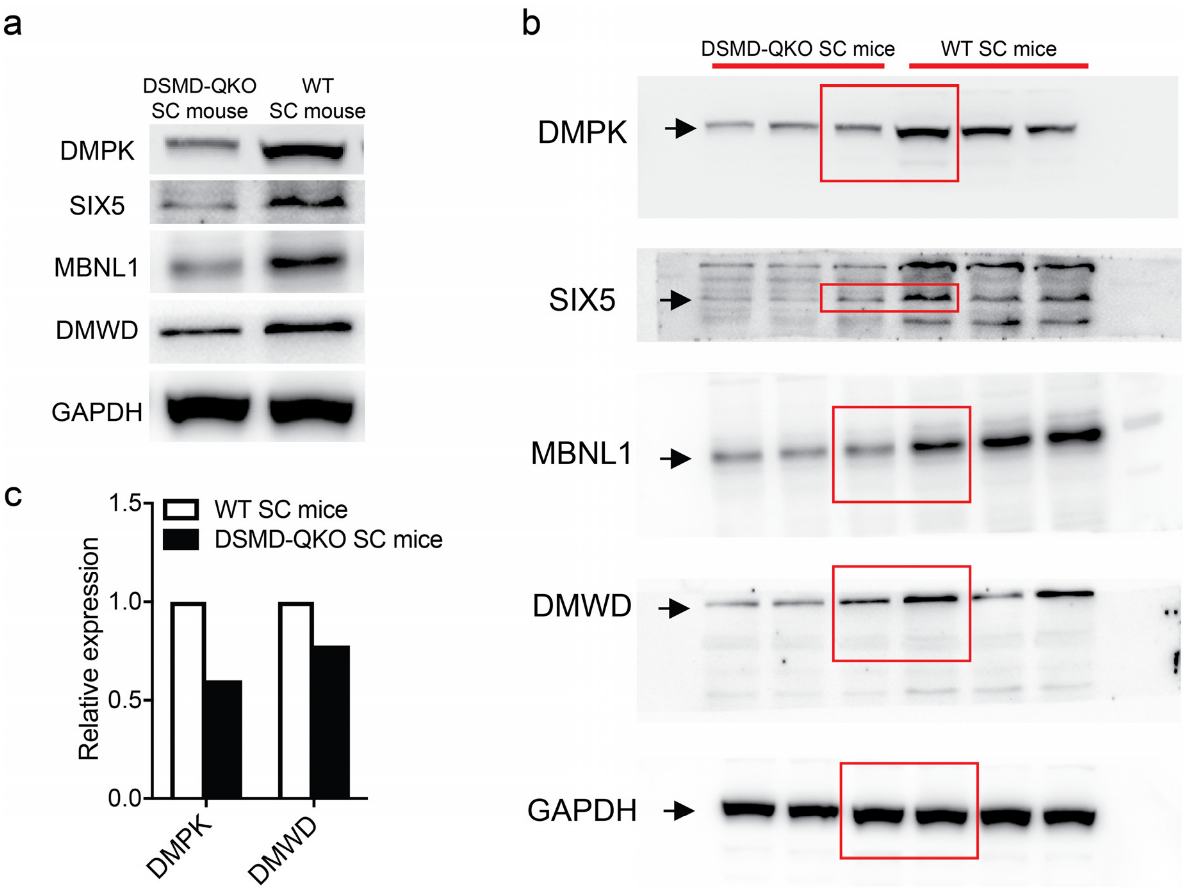

Fig. S6 Protein level analysis of DMPK, SIX5, MBNL1 and DMWD in adult DSMD-QKO SC mice. **a** Western blotting of DMPK, SIX5, MBNL1 and DMWD in the TA muscle of adult QKO and WT SC mice. **b** The original gels of **a**. The red box shown in **a**. **c** Semi-quantitative analysis of the expression of DMPK (stomach) and DMWD (brain) of DSMD-QKO SC mice and WT SC mice using mass spectrometry.
