# Supplementary material for: Dosage effect of multiple genes accounts for multisystem disorder of myotonic dystrophy type 1
Source: Cell Res. 2019 Dec 18;30(2):133–45. doi: 10.1038/s41422-019-0264-2 (PMC7015062; doi:10.1038/s41422-019-0264-2)
Supplement: Supplementary file 7 — Supplementary information, Fig. S7 [file 41422_2019_264_MOESM7_ESM.pdf]

Supplementary information, Figure S7

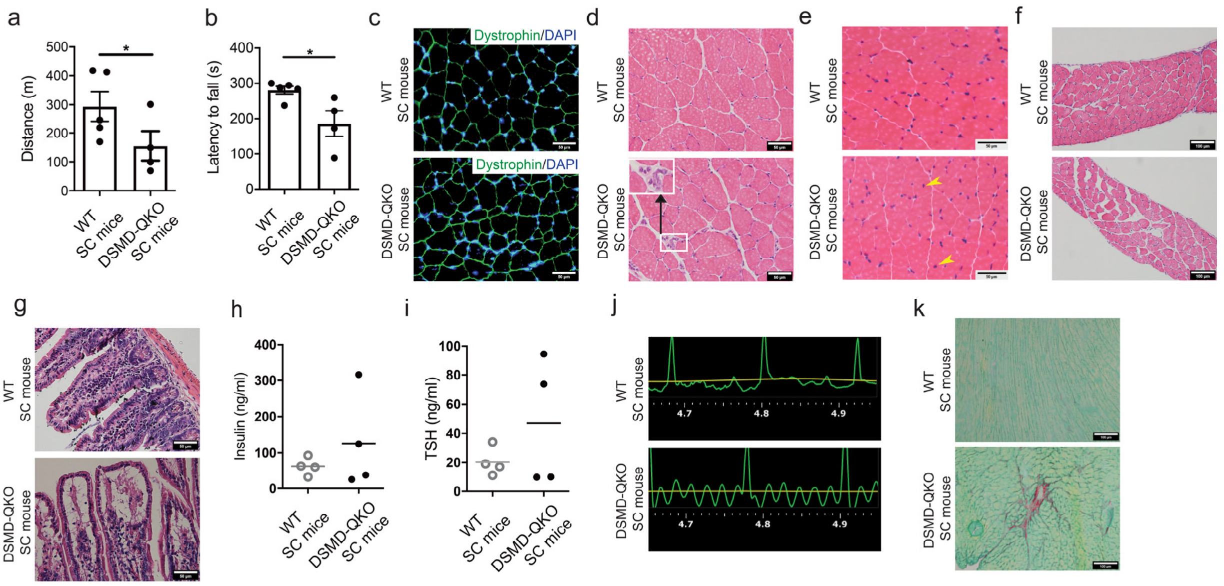

Fig. S7 DM1-associated phenotypes in adult DSMD-QKO SC mice. **a** Treadmill test of QKO SC mice and control SC mice (WT SC mice, $n = 5$; QKO SC mice, $n = 4$, 4-month old). Unpaired Student's t-test, $^*P < 0.05$. **b** Rotarod analysis (WT SC mice, $n = 5$; QKO SC mice, $n = 4$, 4-month old). Unpaired Student's t-test, $^*P < 0.05$. **c** Immunofluorescent staining showing equivalent dystrophin (green) levels in DSMD-QKO and WT SC mice. Scale bars, 50 µm. **d** H&E staining of TA muscle sections from DSMD-QKO and WT SC mice (4-6-month old). White box indicates nuclear clumps in DSMD-QKO muscle. Scale bars, 50 µm. **e** Representative images of myofibers with central nuclei from TA muscle in QKO SC mice (P30). Yellow arrows indicate the myofibers with central nuclei. Scale bars, 50 µm. **f** Histological analysis of diaphragm muscle showing loose construction and increased fat cell invasion in QKO SC mice (4-6-month old). Scale bars, 100 µm. **g** H&E staining showing disorganization of intestine villi in adult DSMD-QKO mice (4-6-month old). Scale bars, 50 µm. **h, i** Insulin and TSH levels determined by ELISA ($n = 4$ per group, 4-6-month old). **j** Electrocardiography experiments showing atrial flutter in DSMD-QKO SC mice (4-6-month old). **k** Representative images of

sirius red staining showing myocardial fibrosis in QKO SC mice (4-6-month old). Scale bars, 100 μm.
