# Supplementary material for: Dosage effect of multiple genes accounts for multisystem disorder of myotonic dystrophy type 1
Source: Cell Res. 2019 Dec 18;30(2):133–45. doi: 10.1038/s41422-019-0264-2 (PMC7015062; doi:10.1038/s41422-019-0264-2)
Supplement: Supplementary file 8 — Supplementary information, Fig. S8 [file 41422_2019_264_MOESM8_ESM.pdf]

Supplementary information, Figure S8

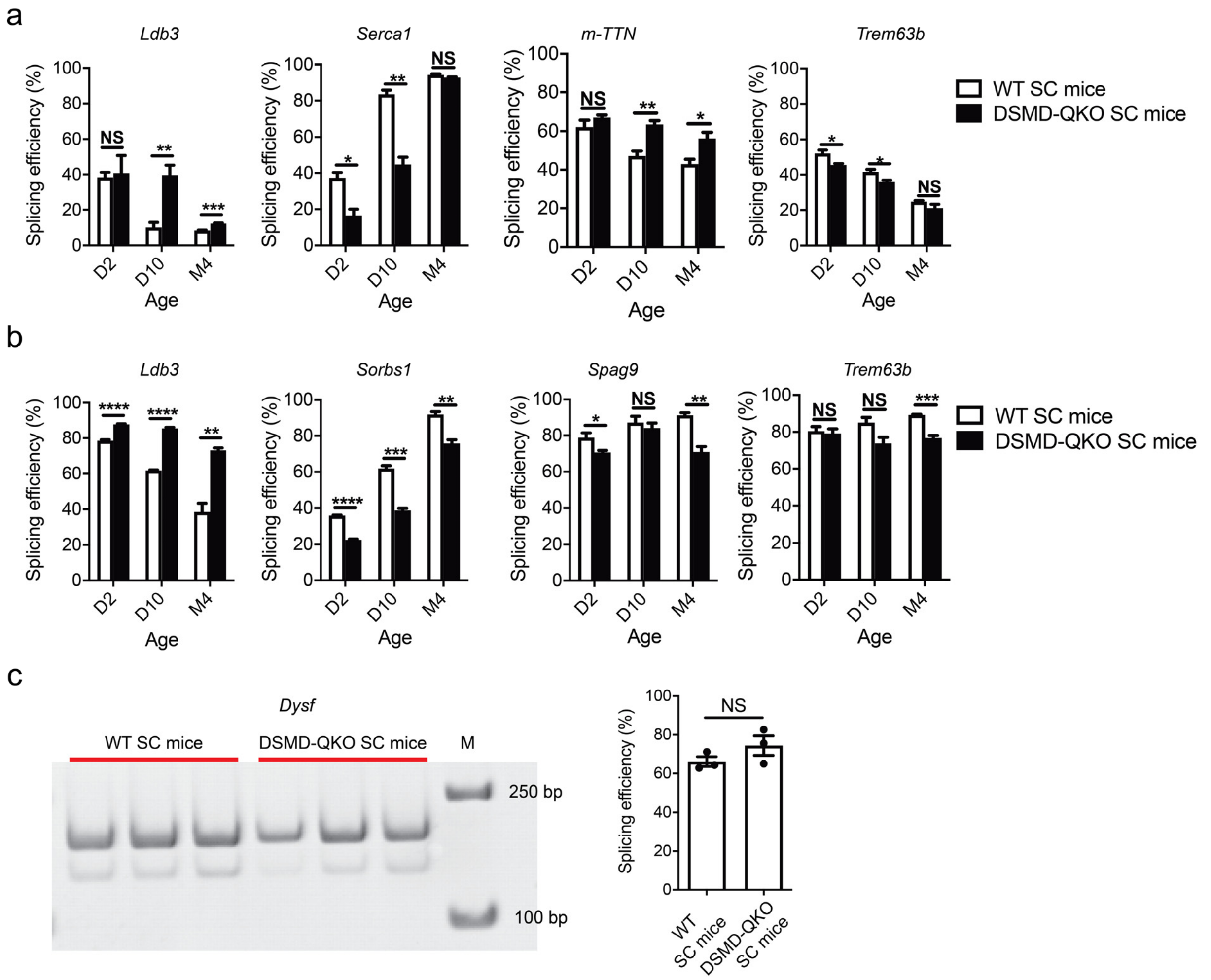

Fig. S8 DM1-associated mis-splicing in DSMD-QKO SC mice. **a** Splicing efficiency of four target pre-mRNAs in TA muscle of QKO and WT SC mice. Unpaired Student's *t*-test, *$P < 0.05$, **$P < 0.01$, ***$P < 0.001$. **b** Splicing efficiency of four target pre-mRNAs in cardiac muscle of QKO and WT SC mice. Unpaired Student's *t*-test, *$P < 0.05$, **$P < 0.01$, ***$P < 0.001$, ****$P < 0.0001$. **c** Splicing efficiency of *Dsyf* that is not changed in muscle of human DM1 patients in TA muscle of DSMD-QKO SC mice and WT SC mice.
