# Supplementary material for: Dosage effect of multiple genes accounts for multisystem disorder of myotonic dystrophy type 1
Source: Cell Res. 2019 Dec 18;30(2):133–45. doi: 10.1038/s41422-019-0264-2 (PMC7015062; doi:10.1038/s41422-019-0264-2)
Supplement: Supplementary file 9 — Supplementary information, Fig. S9 [file 41422_2019_264_MOESM9_ESM.pdf]

Supplementary information, Figure S9

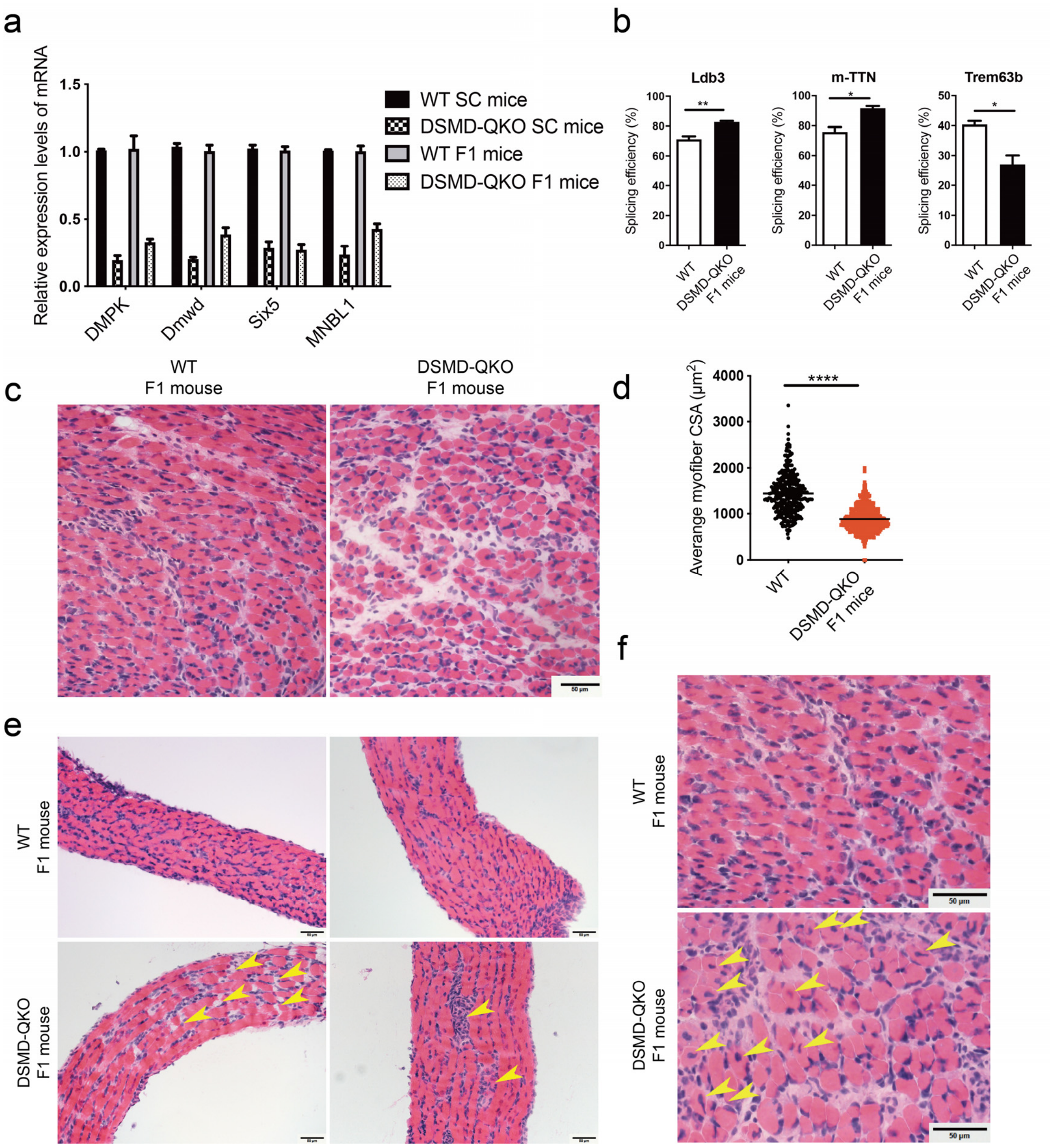

Fig. S9 Phenotype analysis of QKO F1 mice. **a** Relative mRNA expression levels of target genes in QKO SC and F1 mice. **b** Splicing efficiency of three target pre-mRNAs in TA muscle of QKO and WT F1 mice. Unpaired Student's *t*-test, *$P < 0.05$, **$P < 0.01$. **c** H&E staining of TA muscle sections from DSMD-QKO and WT F1 mice (P0). Scale bar, 50 μm. **d** Myofiber CSA analysis in QKO F1 mice (P0) ($n = 3$ per group). Unpaired Student's *t*-test, ****$P < 0.0001$. **e**

Representative images of diaphragm in QKO F1 mice (P0). Yellow arrows indicate nuclear clumps (bottom right) and loose construction (bottom left) in diaphragm. Scale bars, 50 μm. **f** Representative images of myofibers with central nuclei from TA muscle in QKO F1 mice (P0). Yellow arrows indicate the myofibers with central nuclei. Scale bars, 50 μm.
