# Supplementary material for: Dosage effect of multiple genes accounts for multisystem disorder of myotonic dystrophy type 1
Source: Cell Res. 2019 Dec 18;30(2):133–45. doi: 10.1038/s41422-019-0264-2 (PMC7015062; doi:10.1038/s41422-019-0264-2)
Supplement: Supplementary file 10 — Supplementary information, Fig. S10 [file 41422_2019_264_MOESM10_ESM.pdf]

Supplementary information, Figure S10

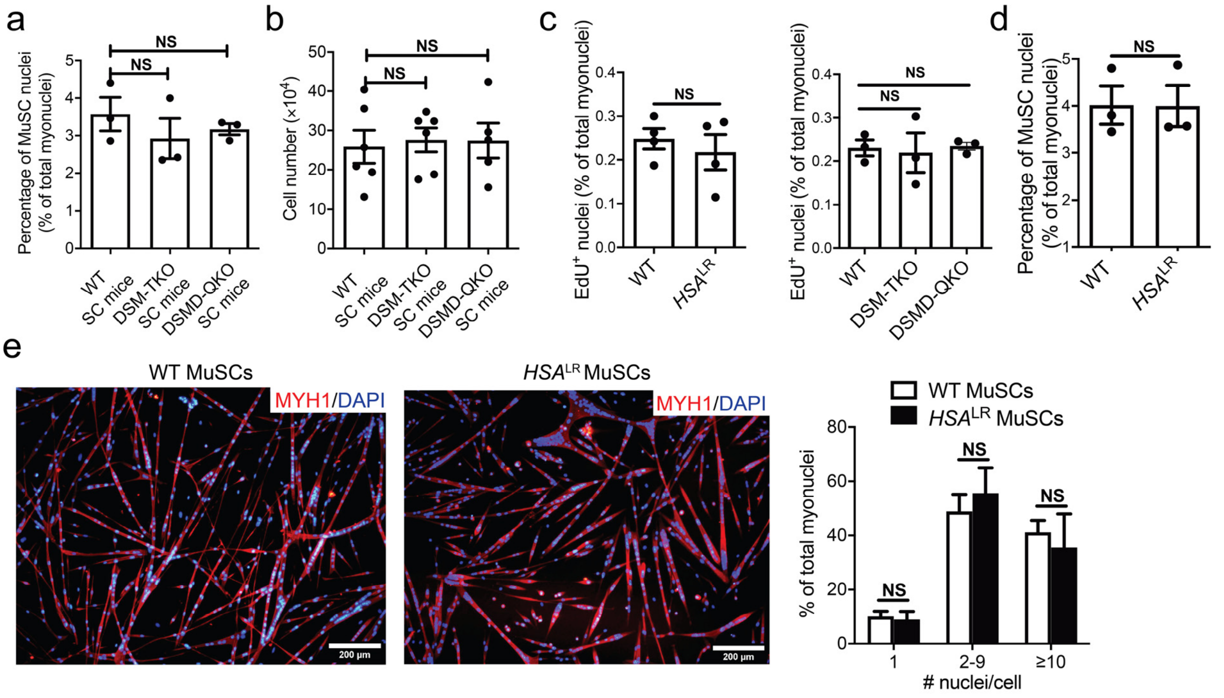

Fig. S10 Normal MuSC number and proliferation in TKO and QKO mice. **a** FACS analysis showing equivalent numbers of MuSCs in TKO, QKO and WT SC mice (n = 3 per group). Unpaired Student's t-test. NS, no significant differences. **b** The proliferation ability of cultured MuSCs (n = 5 or 6 per group). Unpaired Student's t-test. NS, no significant differences. **c** *In vitro* proliferation ability of MuSCs isolated from HSA^{LR} ,TKO, QKO and WT mice (n = 3 or 4 per group). **d** Equivalent number of MuSCs in HSA^{LR} (FVB/n background) and WT mice (FVB/n background) (n = 3 per group). Unpaired Student's t-test. NS, no significant differences. **e** *In vitro* differentiation potential of HSA^{LR} and WT MuSCs. Scale bars, 200 µm. Unpaired Student's t-test. NS, no significant differences.
