# Supplementary material for: Dosage effect of multiple genes accounts for multisystem disorder of myotonic dystrophy type 1
Source: Cell Res. 2019 Dec 18;30(2):133–45. doi: 10.1038/s41422-019-0264-2 (PMC7015062; doi:10.1038/s41422-019-0264-2)
Supplement: Supplementary file 11 — Supplementary information, Fig. S11 [file 41422_2019_264_MOESM11_ESM.pdf]

Supplementary information, Figure S11

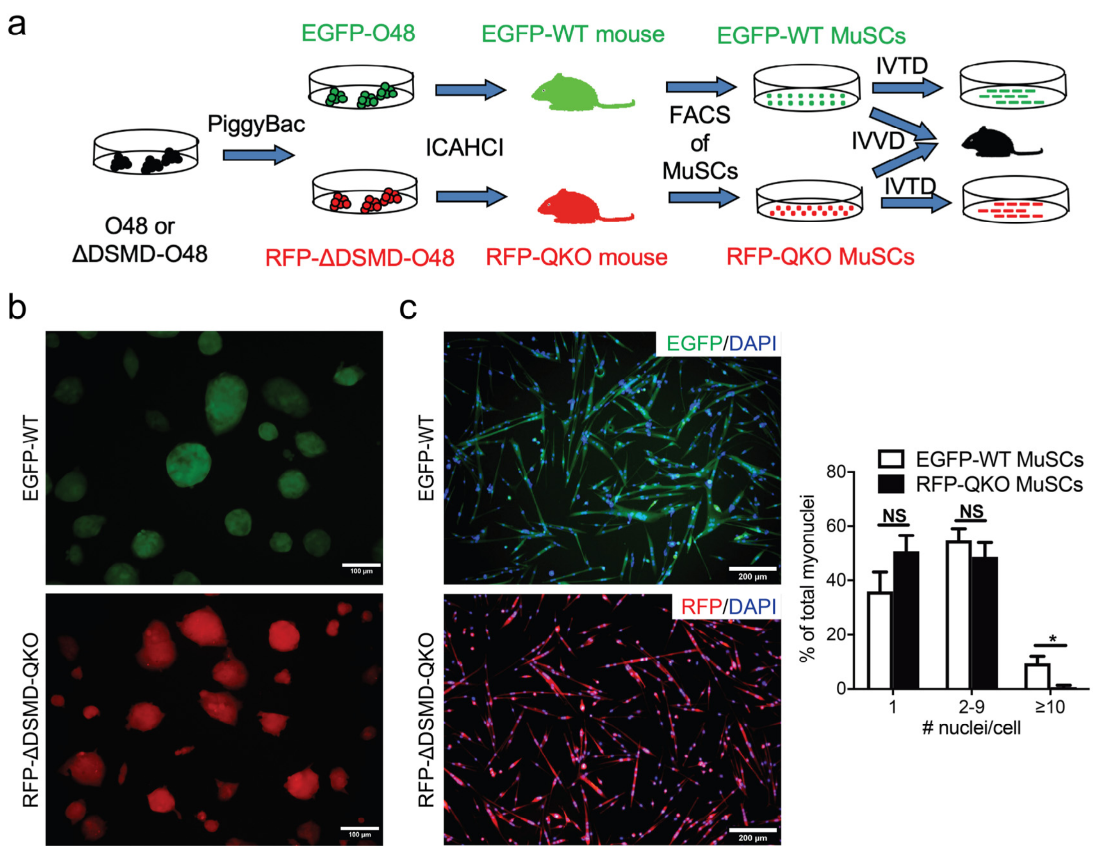

Fig. S11 Differentiation analysis of DSMD-QKO MuSCs. **a** Schematic diagram of *in vivo* differentiation assay of QKO (red fluorescence) and WT (green fluorescence) MuSCs. IVTD, *in vitro* differentiation. IVVD, *in vivo* differentiation. **b** Representative images of RFP-ΔDSMD-O48 and EGFP-O48 haploid ESCs. Scale bars, 100 µm. **c** *In vitro* differentiation of RFP-QKO and EGFP-WT MuSCs. Unpaired Student's *t*-test. *$P < 0.05$. NS, no significant changes. Scale bars, 200 µm.
