# Supplementary material for: Dosage effect of multiple genes accounts for multisystem disorder of myotonic dystrophy type 1
Source: Cell Res. 2019 Dec 18;30(2):133–45. doi: 10.1038/s41422-019-0264-2 (PMC7015062; doi:10.1038/s41422-019-0264-2)
Supplement: Supplementary file 12 — Supplementary information, Fig. S12 [file 41422_2019_264_MOESM12_ESM.pdf]

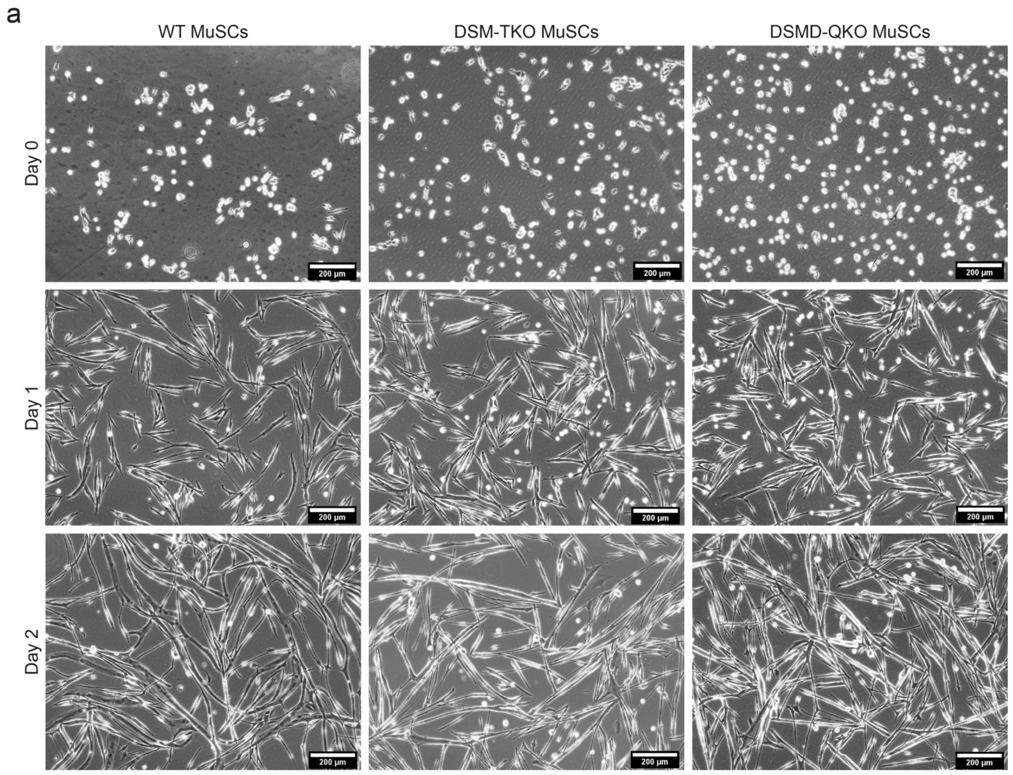

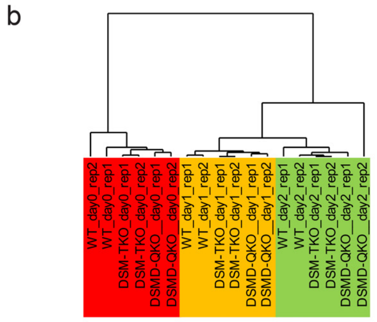

c

Term	PValue
GO:0006936~muscle contraction	6.96E-12
GO:0007275~multicellular organism development	3.18E-09
GO:0086004~regulation of cardiac muscle cell contraction	3.89E-08
GO:0006874~cellular calcium ion homeostasis	1.08E-07
GO:0060048~cardiac muscle contraction	1.98E-07
GO:0007517~muscle organ development	2.25E-07
GO:0006816~calcium ion transport	8.52E-07
GO:0030324~lung development	1.18E-06
GO:0002027~regulation of heart rate	2.44E-06
GO:0006811~ion transport	3.07E-06

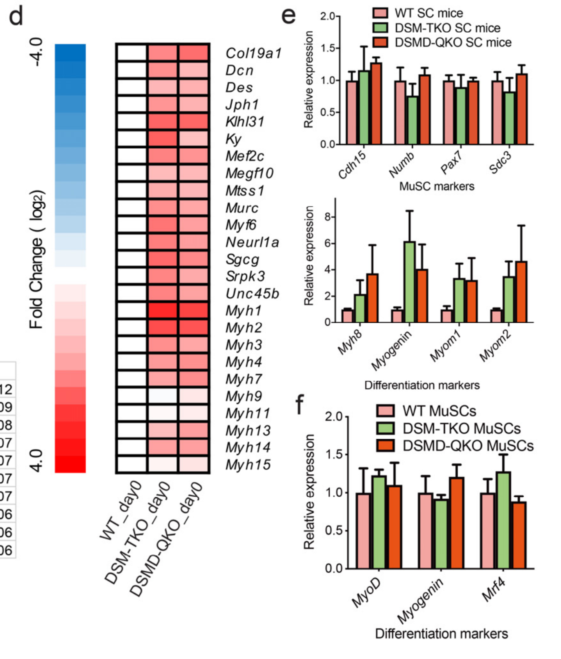

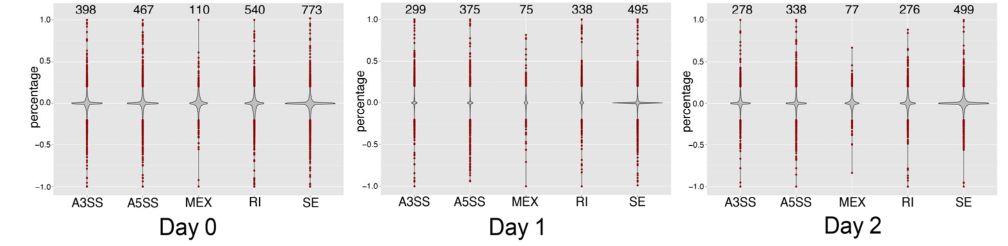

Fig. S12 Reduced stemness in MuSCs of TKO and QKO SC mice. **a** Representative images of MuSCs (Day 0) and cells on the first and second day post differentiation (Day 1 and Day 2) used for RNA-seq analyses. Scale bars, 200 μm. **b** Genomic expression profiles of MuSCs (Day 0) and differentiating cells (Day 1 and Day 2). **c** GO analysis of upregulated genes in TKO and QKO cells compared with WT cells. **d** Heat map analysis of differentiation markers between DM1 MuSCs and WT MuSCs (Day 0). **e** RT-PCR analysis of representative genes of self-renewal and differentiation in MuSCs. The expression values (means ± SD) were normalized to that of *Gapdh*. **f** RT-PCR of differentiation markers in differentiating cells from MuSCs two days post differentiation. **g** Alternative splicing event analysis in MuSCs (Day 0) and differentiating cells (Day 1 and Day 2). A3SS, alternative 3' splice site; A5SS, alternative 5' splice site; MEX, mutually exclusive exons; RI, retained intron; SE, skipped exon.
