# Supplementary material for: Dosage effect of multiple genes accounts for multisystem disorder of myotonic dystrophy type 1
Source: Cell Res. 2019 Dec 18;30(2):133–45. doi: 10.1038/s41422-019-0264-2 (PMC7015062; doi:10.1038/s41422-019-0264-2)
Supplement: Supplementary file 13 — Supplementary information, Table S1 [file 41422_2019_264_MOESM13_ESM.pdf]

Supplementary information, Table S1

Table S1 Development of ICAHCI embryos derived from haploid ESCs with single, triple or quadruple mutations.

Haploid ESC lines used for ICAHCI (the number of cell lines tested)	Passage number	No. of SC embryos transferred (2-cell)	No. of SC pups (% of transferred embryos)	No. of SC pups surviving to P2 (% of birth)	No. of pups surviving to three weeks (% of P2)	No. of pups surviving to adult (% of P2)
O48	P43-P51	430	55 (12.8)	52 (94.5)	52 (100)	50 (96.2)
ΔDmpk-O48 (2)	P41-43	192	26 (13.5)	24 (91.6)	22 (91.6)	21 (87.5)
ΔSix5-O48 (2)	P40	156	15 (9.6)	13 (86.7)	13 (100)	12 (92.3)
ΔMbnl1-O48 (2)	P40-P42	200	19 (9.5)	19 (100)	18 (94.7)	18 (94.7)
ΔDMWD-O48 (2)	P46-47	330	31 (9.4)	30 (96.8)	26 (86.7)	25 (83.3)
DSM-TKO-O48 (2)	P57-P62	270	34 (12.6)	32 (94.1)	30 (93.8)	30 (93.8)
DSMD-QKO-O48 (3)	P66-71	372	45 (12.1)	35 (77.8)	19 (54.3)	17 (48.6)
