# Supplementary material for: Dosage effect of multiple genes accounts for multisystem disorder of myotonic dystrophy type 1
Source: Cell Res. 2019 Dec 18;30(2):133–45. doi: 10.1038/s41422-019-0264-2 (PMC7015062; doi:10.1038/s41422-019-0264-2)
Supplement: Supplementary file 14 — Supplementary information, Table S2 [file 41422_2019_264_MOESM14_ESM.pdf]

Supplementary information, Table S2

Table S2 Off-target analysis of TKO and QKO cells.

sgRNAs of targeted genes	No. of mismatch	No. of found targets (gene)	No. of mutated sites	Cell lines analyzed
Dmpk	0<br>1<br>2<br>3<br>4	1 (*Dmpk*)<br>0<br>1<br>21<br>275	1<br>0<br>0<br>0<br>0	∆DSM-O48-1<br>∆DSMD-O48-2
Six5	0<br>1<br>2<br>3<br>4	1 (*Six5*)<br>0<br>0<br>16<br>186	1<br>0<br>0<br>0<br>0	∆DSM-O48-1<br>∆DSMD-O48-2
Mbnl1-up	0<br>1<br>2<br>3<br>4	1 (*Mbnl1*)<br>0<br>1<br>11<br>220	1<br>0<br>0<br>0<br>0	∆DSM-O48-1<br>∆DSMD-O48-2
Mbnl1-down	0<br>1<br>2<br>3<br>4	1 (*Mbnl1*)<br>0<br>0<br>13<br>198	1<br>0<br>0<br>0<br>0	∆DSM-O48-1<br>∆DSMD-O48-2
Dmwd	0<br>1<br>2<br>3<br>4	1 (*Dmwd*)<br>0<br>4<br>40<br>415	1<br>0<br>0<br>0<br>0	∆DSM-O48-1<br>∆DSMD-O48-2
