# Supplementary material for: Dosage effect of multiple genes accounts for multisystem disorder of myotonic dystrophy type 1
Source: Cell Res. 2019 Dec 18;30(2):133–45. doi: 10.1038/s41422-019-0264-2 (PMC7015062; doi:10.1038/s41422-019-0264-2)
Supplement: Supplementary file 15 — Supplementary information, Table S3 [file 41422_2019_264_MOESM15_ESM.pdf]

Supplementary information, Table S3

Table S3 Clinical features of DM1 and phenotypes in mouse disease models.

	Skeletal muscle				Cardiac problems	Cataract	Endocrine disorders	Digestive system dysfunction	Congenital DM1			Defective satellite cells	References
	Myotonic	Wasting	Weakness	Histology					Hypotonia	Breathing problem	Developmental delay		
Clinical features	+++	+++	++	+++	++	++	++	++	++	++	++	+	[1]
$Dmpk^{-/-}$	-	-	-	+	-	-	ND	ND	-	-	-	ND	[2, 3]
$Six5^{-/-}$	-	-	-	-	-	+	ND	ND	-	-	-	ND	[4, 5]
HSA^{LR} (TG)	+++	-	-	++	-	-	ND	ND	-	-	-	ND	[6]
$Mbnl1^{-/-}$	+++	-	-	++	-	+++	ND	ND	-	-	-	ND	[7]
$Mbnl1^{-/-}$; $Mbnl2^{C/C}$; $Myog$-$Cre^{+/-}$	ND	++	+++	+++	ND	ND	ND	ND	-	+	+	ND	[8]
$Mbnl1^{-/-}$; $Mbnl2^{C/C}$; $Mbnl3^{C/Y}$; $Myog$-$Cre^{+/-}$	ND	+++	+++	+++	ND	ND	ND	ND	+	++	++	ND	[8]
$DMPK^{+/-}$	-	-	ND	-	-	-	-	-	ND	-	-	-	In this study
$SIX5^{+/-}$	ND	-	ND	-	-	-	-	+	ND	-	-	-	In this study
$MBNL1^{+/-}$	ND	-	ND	-	-	-	-	-	ND	-	-	-	In this study
$DMWD^{+/-}$	ND	+	ND	-	-	-	-	-	ND	-	-	-	In this study
DSM-TKO	+	++	++	+	+	-	+	+	-	-	-	+	In this study
DSMD-QKO	+	++	++	+	++	+	+	+	++	++	+	+	In this study

-: No phenotype; +: Mild phenotype; ++: Moderate phenotype; +++: Severe phenotype; ND: No determined.

References

1. Udd, B., and Krahe, R. (2012). The myotonic dystrophies: molecular, clinical, and therapeutic challenges. The Lancet. Neurology *11*, 891-905.
2. Jansen, G., Groenen, P.J., Bachner, D., Jap, P.H., Coerwinkel, M., Oerlemans, F., van den Broek, W., Gohlsch, B., Pette, D., Plomp, J.J., et al. (1996). Abnormal myotonic dystrophy protein kinase levels produce only mild myopathy in mice. Nature genetics *13*, 316-324.
3. Reddy, S., Smith, D.B., Rich, M.M., Leferovich, J.M., Reilly, P., Davis, B.M., Tran, K., Rayburn, H., Bronson, R., Cros, D., et al. (1996). Mice lacking the myotonic dystrophy protein kinase develop a late onset progressive myopathy. Nature genetics *13*, 325-335.
4. Klesert, T.R., Cho, D.H., Clark, J.I., Maylie, J., Adelman, J., Snider, L., Yuen, E.C., Soriano, P., and Tapscott, S.J. (2000). Mice deficient in Six5 develop cataracts: implications for myotonic dystrophy. Nature genetics *25*, 105-109.
5. Sarkar, P.S., Appukuttan, B., Han, J., Ito, Y., Ai, C., Tsai, W., Chai, Y., Stout, J.T., and Reddy, S. (2000). Heterozygous loss of Six5 in mice is sufficient to cause ocular cataracts. Nature genetics *25*, 110-114.
6. Mankodi, A., Logigian, E., Callahan, L., McClain, C., White, R., Henderson, D., Krym, M., and Thornton, C.A. (2000). Myotonic dystrophy in transgenic mice expressing an expanded CUG repeat. Science *289*, 1769-1773.
7. Kanadia, R.N., Johnstone, K.A., Mankodi, A., Lungu, C., Thornton, C.A., Esson, D., Timmers, A.M., Hauswirth, W.W., and Swanson, M.S. (2003). A muscleblind knockout model for myotonic dystrophy. Science *302*, 1978-1980.
8. Thomas, J.D., Sznajder, L.J., Bardhi, O., Aslam, F.N., Anastasiadis, Z.P., Scotti, M.M., Nishino, I., Nakamori, M., Wang, E.T., and Swanson, M.S. (2017). Disrupted prenatal RNA processing and myogenesis in congenital myotonic dystrophy. Genes & development *31*, 1122-1133.
