# Supplementary material for: Dosage effect of multiple genes accounts for multisystem disorder of myotonic dystrophy type 1
Source: Cell Res. 2019 Dec 18;30(2):133–45. doi: 10.1038/s41422-019-0264-2 (PMC7015062; doi:10.1038/s41422-019-0264-2)
Supplement: Supplementary file 16 — Supplementary information, Table S4 [file 41422_2019_264_MOESM16_ESM.pdf]

Supplementary information, Table S4

Table S4 The primers used in this study.

Primers name	Sequence (5'-3')
qPCR	
Cdh15-qPCR-f	GTCACTCAGCCCAATGCTCT
Cdh15-qPCR-r	GAGCAGAGCAGAACCCATTG
Numb-qPCR-f	CTTCCCAGTTAAGTACCTCGGC
Numb-qPCR-r	CCCGTTTTTCCAAAGAAGCCT
Gapdh-qPCR-f	TGTGTCCGTCGTGGATCTGA
Gapdh-qPCR-r	CCTGCTTCACCACCTTCTTGAT
Mrf4-qPCR-f	TGAGGGTGCGGATTTCCTG
Mrf4-qPCR-f	CACGTTTGCTCCTCCTTCCT
MyoD-qPCR-f	GGCTACGACACCGCCTACTA
MyoD-qPCR-r	GTGGAGATGCGCTCCACTAT
Myogenin-qPCR-f	GAGACATCCCCCTATTTCTACCA
Myogenin-qPCR-r	GCTCAGTCCGCTCATAGCC
Myh8-qPCR-f	GGAGAGGATTGAGGCCCAAAA
Myh8-qPCR-r	CACGGTCACTTTCCCTCCATC
Myom1-qPCR-f	GCACGACCATGAGCCACTAC
Myom1-qPCR-r	ACCCTTGAGAATGCCGGGA
Myom2-qPCR-f	AAAAGACACAAGCACTTTGACCA
Myom2-qPCR-r	TGGGAGGATGACTGGGTGG
Pax7-qPCR-f	GACGACGAGGAAGGAGACAA
Pax7-qPCR-r	ACATCTGAGCCCTCATCCAG
Sdc3-qPCR-f	AGAGGCCGGTGGATCTTGA
Sdc3-qPCR-r	CTCCTGCTCGAAGTAGCCAGA
CRISPR-Cas9 targeting sequence	
Dmpk-sgRNA-f	CACCGCCCATTGCAGCAAGGCTTA
Dmpk-sgRNA-r	AAACTAAGCCTTGCTGCAATGGGC
Six5-sgRNA-f	CACCGTCTGATGGGAACCCCACTA
Six5-sgRNA-r	TAGTGGGGTTCCCATCAGAC
Mbnl1-up-sgRNA-f	CACCGCTGCAACACTATCTGTGAT
Mbnl1-up-sgRNA-r	AAACATCACAGATAGTGTTGCAGC
Mbnl1-down-sgRNA-f	CACCGGTACATGGTACTCTAAATT
Mbnl1-down-sgRNA-r	AAACAATTTAGAGTACCATGTACC
Dmwd-sgRNA-f	CACCGGCTTCTACAAGCTGCTCCC
Dmwd-sgRNA-r	AAACGGGAGCAGCTTGTAGAAGCC
Knockout mouse genotyping	
Dmpk-test-f	CACAGCTCCAGTTCGAGGTT
Dmpk-test-r	AGATGACAGCAAGAGGTGGC
Six5-test-f	CCCAGCAGGCTTGTAGAGAC
Six5-test-r	GCCAGGAAGCTCCCATTCAC

Mbnl1-test-f	AGAACCAGAGGGAAAGCAGC
Mbnl1-test-r	GAGGCATGGAGTTCGGAGTT
Mbnl1-wt-f	CAGCCTCTGCTTTTGCCTTG
Mbnl-wt-r	CGTGTGCTCAGTAACCCCTT
Dmwd-test-f	CGCTGTGTGATCGTGTGCAT
Dmwd-test-r	TGGTGTCTTTCTTGATGAGGTC
Splicing analysis	
Ldb3-sp-f	GCGTCAACACAGACACCATGACC
Ldb3-sp-r	TCATCTGGGCCAGGATGCGGAA
Serca1-sp-f	GCTCATGGTCCTCAAGATCTCAC
Serca1-sp-r	GGGTCAGTGCCTCAGCTTTG
m-TTN-sp-f	GTGTGAGTCGCTCCAGAAACG
m-TTN-sp-r	CCACCACAGGACCATGTTATTTC
Trem63b-sp-f	CTGGCTCTGGACTTCATGTGCTTTC
Trem63b-sp-r	GAGACGGAGGTGAGACGCTCATACC
Sorbs1-sp-f	CCAGCTGATTACTTGGAGTCCACAGAAG
Sorbs1-sp-r	GTTCACCTTCATACCAGTTCTGGTCAATC
Spag9-sp-f	GGACTGGAAATGGTGTCATTATCTCCAT
Spag9-sp-r	GGGACTGCCACAAAGAATTTCACAG
Dysf-sp-f	CGAAAATCTCTGCTACTGGAGGAG
Dysf-sp-r	TGTGTTGAGCTCTGCATAAGGATC
Mbnl1-sp-f	ACACCTGCAAGCCAAGATCA
Mbnl1-sp-r	TGTTAAAGACTGCGGTGGCA
HSA^{LR} mice genotype	
MSA-f	TCCTCAGGACGACAATCGAC
MSA-r	CCTAAGGAGTTCACCCAGTCTG
HSA-f	AAACTTACATCTTCCCATGCTCC
HAS-r	GAGACGCCCTCTGAGAAACAG
